# Physical fitness and clinically assessed disease burden in long‐term childhood cancer survivors—The SURfit study

**DOI:** 10.1002/cncr.70051

**Published:** 2025-08-19

**Authors:** Anna K. Mayr, Simeon Zürcher, Iris Bänteli, Helge Hebestreit, Rahel Kasteler, Nicolas X. von der Weid, Susi Kriemler, Christina Schindera, Corina S. Rueegg

**Affiliations:** ^1^ Department of Medicine University of Zurich Zurich Switzerland; ^2^ Center for Psychiatric Rehabilitation Universitäre Psychiatrische Dienste Bern (UPD) and University Hospital of Psychiatry and Psychotherapy University of Bern Bern Switzerland; ^3^ Department of Psychosomatic Medicine University Hospital and University of Basel Basel Switzerland; ^4^ Pediatric Department University Hospital Julius‐Maximilians University Würzburg Germany; ^5^ Department of Oncology, Hematology, Immunology Stem Cell Transplantation and Somatic Gene Therapy University Children’s Hospital Zurich Zurich Switzerland; ^6^ Department of Pediatric Hematology and Oncology University Children’s Hospital Basel (UKBB) and University of Basel Basel Switzerland; ^7^ Epidemiology, Biostatistics and Prevention Institute University of Zurich Zurich Switzerland; ^8^ Childhood Cancer Research Group Institute of Social and Preventive Medicine University of Bern Bern Switzerland; ^9^ Oslo Centre for Biostatistics and Epidemiology Oslo University Hospital Oslo Norway

**Keywords:** adverse events, aerobic capacity, childhood cancer survivors, clinical assessment, Common Terminology of Adverse Events, disease burden, physical fitness

## Abstract

**Background:**

Identifying disease burden among childhood cancer survivors (CCS) can guide tailored care. Physical fitness predicts health and mortality and may help reduce disease burden in CCS. This study aimed to 1) describe the burden of clinically ascertained adverse health outcomes in long‐term CCS, and 2) investigate the association between physical fitness and adverse health outcomes.

**Methods:**

This study used baseline data of the SURfit study, a randomized controlled physical activity trial. The authors included 163 CCS, diagnosed <16 years, ≥16 years at enrollment, and ≥5 years since last cancer diagnosis. Clinically assessed health outcomes were categorized using the Common Terminology Criteria for Adverse Events. Physical fitness was assessed by cardiopulmonary‐exercise‐test (CPET), hand‐grip strength, and the 1‐minute sit‐to‐stand test (STS). Using multivariable Poisson regression models, this study investigated the association between physical fitness and adverse health outcomes.

**Results:**

Participants (30.5 ± 8.6 years old, time since diagnosis 22.9 ± 9 years) had 1170 adverse health outcomes, with 99% CCS having at least one. Musculoskeletal disorders were most common (130 of 163 [80%]). Higher levels of physical fitness were associated with fewer adverse health outcomes of any grade (CPET: prevalence rate ratio [PRR], 0.71 per watt/kg bodyweight, 95% confidence Interval [CI], 0.63–0.81, *p* < .001; hand‐grip: PRR, 0.60 kg/kg bodyweight; 95% CI, 0.35–1.03, *p* = .063; STS: PRR, 0.95 per five repetitions; 95% CI, 0.93–0.97, *p* < .001).

**Conclusion:**

CCS participating in an exercise intervention trial experienced a high burden of adverse health outcomes. Increased physical fitness was associated with reduced disease burden for all survivors, emphasizing the importance of encouraging fitness improvements, regardless of cancer history.

## Abstract 2

Childhood cancer survivors face a significant burden of adverse outcomes long after diagnosis, with musculoskeletal disorders being the most common. Higher physical fitness is associated with a reduced prevalence of adverse outcomes for all survivors, regardless of their cancer history.

## INTRODUCTION

Advances in multimodal therapy and supportive care^1^ have significantly increased survival rates among childhood cancer patients, resulting in 5‐year survival exceeding 85% in developed countries.^2^ In this growing population of childhood cancer survivors (CCS), the focus of care shifts beyond cure to encompass long‐term physical and psychosocial health^3^ as CCS are at risk of adverse health outcomes with increased morbidity and mortality.^4^ These adverse health outcomes reflect an aging phenotype driven by the long‐term impact of anticancer therapies.^5^ A study from St. Jude Children’s Research Hospital reported that by the age of 45 years, up to 95% of CCS experienced at least one adverse health outcome, with 81% facing a serious or life‐threatening one.^6^ Multimorbidity has become a major concern in this population, as it is associated with poor quality of life^7^, ^8^ and increased health care costs.^9^, ^10^

Physical fitness is a strong predictor of health and mortality^11^, ^12^, ^13^ and includes body composition, muscle strength, flexibility, and cardiopulmonary fitness. As childhood cancer survivors have lower physical fitness compared to the general population this may further contribute to their overall disease burden.^14^, ^15^, ^16^, ^17^, ^18^ Physical fitness may not affect survivors in the same way as it does their healthy peers, likely due to treatment‐related damage to the cardiovascular and muscular systems.^19^ Understanding the relationship between physical fitness and disease burden is therefore crucial to inform survivorship care, counselling, and targeted physical activity interventions.

Compared to little possibilities to modify cancer treatment, fitness can be improved through lifestyle interventions.^17^, ^20^, ^21^, ^22^, ^23^ Yet, most lifestyle intervention studies are US‐based, where oncologic treatment guidelines and lifestyle differ from European standards,^24^, ^25^, ^26^ leaving a knowledge gap in health burdens and potential modifiable risk factors for European CCS. Babecoff et al.^27^ performed one of few studies that assessed health outcomes in European survivors with grading by severity, however, they did not establish associations to physical fitness. Northern countries with national cancer registries have published large multicenter studies,^28^ however, these either focused on mortality only,^4^, ^29^ relied on self‐reporting (questionnaires),^30^, ^31^ or were assessed on hospitalization data only.^32^ None of these studies draw associations to any fitness component. Thus, our study is the first European Childhood Cancer Survivor Study relating health outcomes to fitness components.

This study aimed to 1) describe the burden of clinically ascertained adverse health outcomes in long‐term survivors of childhood cancer, and 2) investigate the association between physical fitness and prevalence of health outcomes, before and after controlling for cancer‐related variables.

## MATERIALS AND METHODS

### Study design

This study is based on data collected during the baseline assessment of the SURfit study (https://ClinicalTrials.gov/study/NCT02730767). The SURfit study is a single‐center, randomized controlled trial (RCT) with a 1‐year physical activity intervention that has been described before.^33^, ^34^, ^35^, ^36^, ^37^, ^38^, ^39^ In short, participants were recruited through the Swiss Childhood Cancer Registry (ChCR) between 2015 and 2019. Eligible survivors had been diagnosed with cancer according to the International Classification of Childhood Cancer third edition (ICCC‐3)^40^ or with Langerhans cell histiocytosis, diagnosed and/or treated in one of four centers of the Swiss Pediatric Oncology Group, <16 years old at diagnosis, ≥16 years old at enrollment, and ≥5 years since last cancer diagnosis (first diagnosis, relapse, or secondary cancer[s]). The study was approved by the ethics committee of Northwest and Central Switzerland (BASEC‐ID: 2019‐00410), and all participants gave their written informed consent before study enrollment. Participation of minors required consent of their guardians.

### Assessment of adverse health outcomes

Participants underwent extensive medical examinations at the University Children’s Hospital Basel including assessing patient history, clinical examinations, blood analyses, physical fitness testing, electrocardiogram (ECG), oral glucose tolerance test, and dual energy x‐ray absorptiometry (DXA).^39^ Outcomes by DXA included body composition and total body, femoral neck, total hip, and lumbar spine areal bone‐mineral‐density by age‐ and sex‐matched *z* scores, in line with our previous publications.^36^ All adverse health outcomes that occurred after the first cancer diagnosis until baseline assessment of this study were classified according to the Common Terminology Criteria of Adverse Events (CTCAE) version 5^41^ and supplemented by a specified SOP (Appendix S1) using system organ class (SOC) and preferred term (PT). We retained the original categories and names for all SOC, with the following exceptions: “cardiac” and “vascular” disorders were combined into one SOC “cardiovascular disorder”; “general disorders and administration site conditions” were renamed “general symptoms: pain, fatigue, edema”; and “investigations” was renamed “laboratory, imaging, and functional findings.” Each adverse event was graded from 1 to 4 (grade 1, mild, requiring observation only; grade 2, moderate, with minimal or noninvasive intervention indicated, but limiting instrumental Activities of Daily Life [ADL] possible; grade 3, severe, with limited ability of self‐care ADL; grade 4, life‐threatening requiring urgent intervention). Grade 5, death, was not applicable in this study. Whenever no CTCAE criteria were available, predefined criteria were applied (see Appendix S1). Although each CTCAE event was allocated to one specific grade, each patient could qualify for multiple events. Mental health and fatigue was self‐reported using the standardized and validated Brief Symptom Inventory (BSI‐53)^42^ and Checklist of Individual Strength (CIS).^43^ According to CTCAE, the grade 1 event depression and anxiety is defined as “mild depressive/anxiety symptoms,” hence self‐reported depressive/anxiety symptoms in the BSI were classified as such. Patients with officially diagnosed depression/anxiety were classified as grade 1 if not treated and grade 2 if receiving treatment (medical or psychotherapy).

### Assessment of physical fitness

Physical fitness components were defined according to our previous publication^37^ and described in detail in our protocol publication (for detail see Appendix S1).^39^ Aerobic capacity was assessed by means of a cardiopulmonary exercise test (CPET) and results expressed as peak work rate in watt per kilogram bodyweight (watt/kg bodyweight). Peak performance (watt) was assessed by a continuous incremental cycling test to volitional exhaustion following a validated protocol.^44^ Upper body strength was assessed by hand‐grip strength test with a JAMAR hydraulic hand dynamometer. We calculated the mean weight of three trials in kilograms reached by the dominant hand^45^ per kilogram of bodyweight (kg/kg bodyweight). Lower body endurance was assessed by a 1‐min sit‐to‐stand (STS) test. The number of repetitions was divided by 5 (the estimated minimal important difference^46^) to have a meaningful unit in the model.

We additionally calculated *z* scores for each fitness variable based on age and sex‐stratified normative populations.^45^, ^47^, ^48^ We then calculated a composite fitness score averaging the *z* scores of the three fitness measures. If participants had a valid result of at least two out of three measures the average over the available measures was calculated.

### Covariates

Information on age at study, sex, sociodemographic factors, and cancer history was collected. Time since cancer diagnosis was calculated as time from first cancer diagnosis to date of baseline assessment in years. Cancer diagnosis was recorded according to ICCC‐3 and aggregated into five groups: leukemias, lymphomas, central nervous system (CNS) tumors, bone‐ and soft tissue sarcomas, and other tumors. We calculated the cumulative anthracyclines dose as doxorubicin isotoxic equivalent dose (mg/m^2^)^49^ and the cumulative steroid dose as prednisone equivalent dose (mg/m^2^).^50^ Radiotherapy was grouped into no radiotherapy, cranial, abdominal, total body irradiation (TBI), or other location. Body weight and height were measured, body mass index (BMI) calculated (weight in kg/height in m^2^), and allocated to four categories (kg/m^2^) according to the World Health Organization (WHO)^51^: underweight (<18.5), normal weight (18.5–24.9), overweight (25–29.9) and obese (≥30).

### Statistical analysis

For descriptive statistics, we used mean, range and standard deviation (SD) and ranges, medians with ranges, or frequencies with proportions, as appropriate. We described the cumulative burden of adverse events (aim 1) by reporting total number of events, frequency, and percentage of survivors with at least one event, and average number of events per person by SOC and PT. All results were displayed for events of any grade (grades 1–4), grades 2+ (grades 2–4), and grades 3+ (grades 3–4).

We investigated the association between physical fitness and adverse health outcomes (aim 2) by applying multivariable Poisson regression models to estimate prevalence rate ratios (IRRs) with 95% confidence intervals (CIs). We performed a separate model for each physical fitness parameter (aerobic capacity, hand‐grip strength, lower body endurance, and composite fitness score) and outcome (any grade, grades 2+, and grades 3+). Model adjustments for each association were based on a directed acyclic graph (Figure S1). Model 0 included only age, sex, and time since diagnosis (to condition on the same time interval when calculating IRRs). Model 1 additionally adjusted for BMI (categorical), and model 2 additionally included cancer‐related variables (cancer diagnosis, cumulative anthracycline dose, cumulative steroid dose, and location of radiotherapy). For comparison of estimates across the fitness parameters, we performed the same models using *z* scores instead of absolute values. Participants missing a physical fitness parameter were excluded from the respective models. We performed a sensitivity analysis adjusting the fitness parameters for lean body mass (LBM).

For our main analysis, we included all CPET results (maximal and submaximal) for the association between aerobic capacity and adverse health outcomes. To investigate possible bias, we performed a sensitivity analysis including only maximal tests (*n* = 128). All analyses were performed using STATA SE v18.0, and a *p* value <.05 was considered statistically significant.

## RESULTS

### Participant characteristics

**FIGURE 1:**
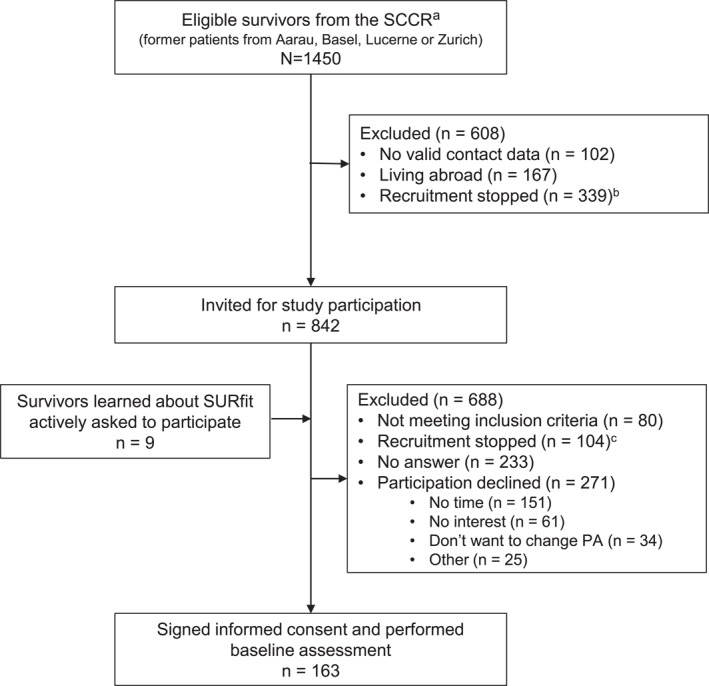
Flowchart of sample inclusion. ^a^Eligible survivors were identified in the population‐based SCCR. ^b^Recruitment was stopped due to participant saturation. ^c^Participants who received an initial invitation were no longer followed up by phone due to participant saturation. PA indicates physical activity (intervention after baseline assessment); SCCR, Swiss Childhood Cancer Registry.

Of 1450 eligible study participants identified in the ChCR, 842 met the inclusion criteria and were invited to participate in the SURfit study; 163 CCS underwent the baseline assessment and were included in the analysis (Figure 1). At study entry, participants were on average 30.5 years of age (SD, 8.6) with a range from 17 to 49 years old (Table 1). Mean time since diagnosis was 22.9 years (SD, 9.0). Most survivors had leukemia (*n* = 57, 35%), were of normal weight (*n* = 102, 63%), had received chemotherapy (*n* = 148, 91%), and nearly half of survivors had received radiotherapy (*n* = 96, 59%) as part of their cancer treatment.

**TABLE 1:** Characteristics of survivors included in the study (SURfit study, *N* = 163).

Characteristics	
Age at study in years	30.5 ± 9 [17–49]
Sex, No. (%)	
Male	91 (56)
Female	72 (44)
Height, cm	
Male	176.3 ± 7.3 [153–193]
Female	163.0 ± 6.9 [144–181]
Weight, kg	
Male	76.1 ± 15.1 [42–137]
Female	62.4 ± 11.3 [37–90]
BMI, No. (%)	
Underweight (<18.5 kg/m^2^)	7 (4)
Normal (18.5–24.9 kg/m^2^)	102 (63)
Overweight (25–29.9 kg/m^2^)	40 (25)
Obese (≥30 kg/m^2^)	14 (9)
Lean body mass (kg)	48.5 ± 10.2 [27–72]
Fat mass (kg)	22.1 ± 6.7 [9–38]
Fat percentage (%)	31.2 ± 7.1 [17–53]
Primary cancer diagnosis, No. (%)	
Leukemias	57 (35)
Lymphomas	35 (21)
CNS tumors	18 (11)
Bone tumors and soft tissue sarcomas	19 (12)
Other tumors	34 (21)
Second primary neoplasm, No. (%)^a^	
No	154 (94)
Yes	9 (6)
Received surgery, No. (%)	
No surgery	68 (42)
1 surgery	70 (43)
≥2 surgeries	25 (15)
Received chemotherapy, No. (%)	
No	15 (9)
Yes	148 (91)
Received anthracyclines	106 (65)
Cumulative anthracycline dose^b^ (mg/m^2^)	240 [45–670]
Received steroids	93 (57)
Cumulative steroid dose^c^ (mg/m^2^)	3410 [720–17720]
Received radiotherapy, No. (%)	
No radiotherapy	96 (59)
Cranial	28 (17)
GH deficiency^d^	5 (18)
No GH deficiency	23 (82)
Abdominal	14 (9)
Total body	6 (4)
Other	19 (12)
Received hematopoietic stem cell transplantation, No. (%)^e^	
No	153 (94)
Yes	10 (6)

*Note*: Numbers are presented as mean ± standard deviation [minimum–maximum], median [minimum–maximum], or frequency (percentage).

Abbreviations: BMI, body mass index; CNS, central nervous system; gh, growth‐hormone.

^a^ Does not include relapse(s) or metastasis.

^b^ Doxorubicin isotoxic equivalent dose.

^c^ Prednisone equivalent dose. Six participants who received steroids had missing information on cumulative steroid dose.

^d^ Further two patients that did not receive cranial radiation had GH deficiency (i.e. total *n* = 7 with GH deficiency); all seven patients received GH substitution.

^e^ Autologous (*n* = 3) or allogenic (*n* = 7).

### Number of adverse health outcomes

**FIGURE 2:**
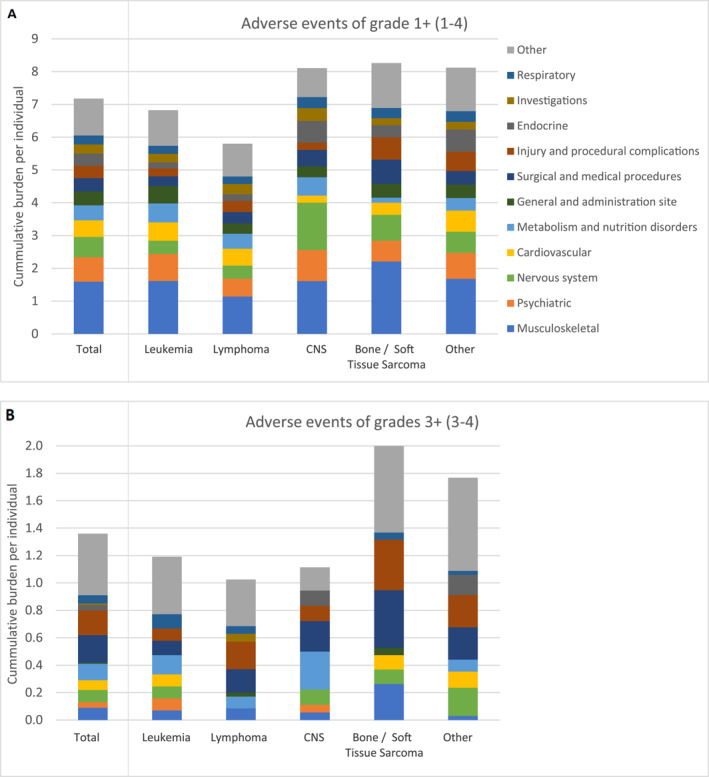
Average number of adverse events per individual grouped by SOC, overall (total), and by cancer diagnosis. Average number of events per persons of (A) any grade (1–4) and (B) grades 3+ (3–4) grouped by SOC according to the CTCAE. The total bar combines all primary cancer diagnosis, hence represents an average across all tumor types. The stacked bars show the respective share each SOC contributes to the total individual burden; SOCs that contributed on average <0.25 events/person were combined into “Other.” The full official name of each SOC is given in Table S1. The legend is ordered (from bottom to top) according to the decreasing average number of events from the total bar in (A) (events of any grade) and kept consistent throughout all bars for comparability. The “Other” group was added at the end of the legend/top of each bar. CNS indicates central nervous system; CTCAE, common terminology criteria for adverse events; SOC, system organ class.

We recorded a total of 1170 adverse health outcomes (any grade) among the 163 participants (Table S1), with 99% of participants having at least one adverse health outcome (only one participant had no event). The maximum number of outcomes per survivor was 27. On average, participants had 7.2 (SD, 4.5) adverse health outcomes (any grade) and 1.4 (SD, 1.6) severe health outcomes (grades 3+) (Figure 2). Survivors of childhood lymphoma had the least average events of any grade per person (5.8, SD, 3.2), whereas CNS‐ and bone‐tumor survivors had the most (8.2, SD, 3.6 and 8.2, SD, 4.2, respectively). Severe adverse events (grades 3+) showed a similar pattern: lymphoma survivors had on average 1.0 (SD, 1.2), CNS‐tumor survivors 1.1 (SD, 1.7), and bone‐tumor survivors 2.0 (SD, 2.0) adverse health outcomes per person (Figure S2). The most common severe health outcomes were injury and procedural complications (15% of participants), followed by complications during or requiring surgical and medical procedures (15%).

### Types of adverse health outcomes

**FIGURE 3:**
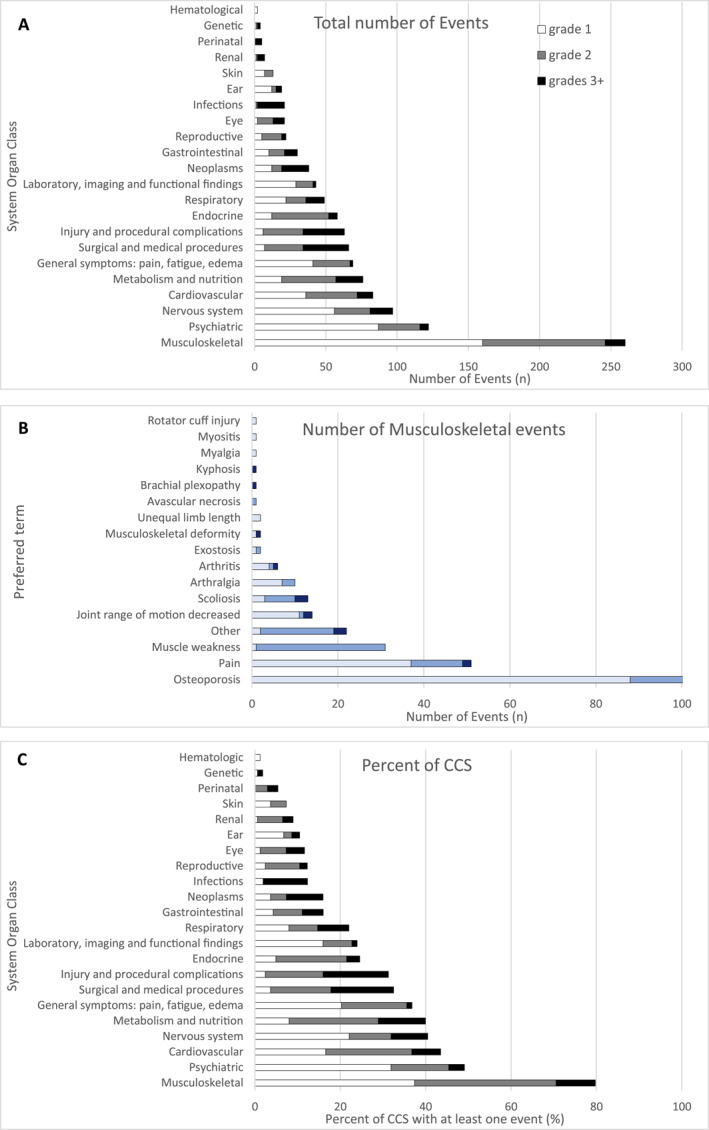
Adverse events in the study population of CCS according to different categorization. The total number of events by all participants (*n* = 163) of (A) any SOC and (B) for the subgroups (PT) of SOC “Musculoskeletal and connective tissue disorders” according to the CTCAE, categorized into grade 1, grade 2, and grades 3+ (3–4). In (B), variables that describe the same symptom, but are defined as different PT by CTCAE are summarized into their overarching symptom: pain includes “back pain,” “neck pain,” and “pain in extremities”; muscle weakness includes “general muscle weakness,” “muscle weakness left/right side,” “muscle weakness trunk,” and “muscle weakness upper limb”; joint range of motion decreased also includes “joint range of motion decreased lumbar spine.” (C) Display of the proportion of CCS with at least one event in the respective SOC, categorized into grades. All panels are ordered by descending frequency/percentage. The full official name of each SOC is given in Table S1. CCS indicates childhood cancer survivors; CTCAE, common terminology criteria for adverse events; PT, preferred terms; SOC, system organ class.

Musculoskeletal disorders were most common across all cancer types (260 outcomes, 80% of participants) (Figure 3A,C) followed by psychiatric conditions (122 events, 49% of participants), nervous system disorders (97 events, 41% of participants), and cardiovascular disorders (83 events, 44% of participants). Osteoporosis was the most frequent musculoskeletal disorder (101 of 163 [62%]) followed by pain (51 of 163 [31%]) and muscle weakness (31 of 163 [17%]). Insomnia (67 of 163 [41%]), depressive symptoms (grade 1+, 28 of 163 [17%]), and depression requiring therapy (grade 2+, 7 of 163 [4%]) were the most frequent psychiatric conditions (Figure 3B; Table S2). Survivors of CNS‐tumors had notably more nervous system disorders (16 of 18 [89%]) and ear and labyrinth disorders (4 and 18 [22%]) (Figure S3). Injury and procedural disorders were more prominent among bone cancer survivors (11 of 19 [58%]) with fractures being the most frequent PT (42 of 63 [67%]). Tables S1 and S2 provide further detailed information on all registered adverse events by SOC and PT.

### Physical fitness and adverse health outcomes

Higher physical fitness was associated with lower prevalence of adverse health outcomes across all fitness parameters and grades of outcomes, reaching statistical significance in almost all models except for hand‐grip strength (Table 2). The strength of associations did not substantially change when adjusting for cancer variables. In the fully adjusted models (including cancer variables), increased aerobic capacity (per watt/kg bodyweight) was significantly associated with lower prevalence of events of any grade (PRR = 0.71; 95% CI, 0.63–0.81, *p* < .001), grades 2+ (PRR = 0.71; 95% CI, 0.59–0.84, *p* < .001), and grades 3+ (PRR = 0.64; 95% CI, 0.47–0.86, *p* = .003). Increased lower body endurance (per five repetitions more in the 1 min STS) was significantly associated with lower prevalence of events of any grade (PRR = 0.95; 95% CI, 0.93–0.97, *p* < .001), grades 2+ (PRR = 0.95; 95% CI, 0.92–0.98, *p* = .003), and grades 3+ (PRR = 0.92; 95% CI, 0.87–0.98, *p* = .002).

**TABLE 2:** Association between physical fitness and prevalence of adverse events from Poisson regression models (*N* = 159–163)^a^

	Basic model 0^b^			Adjusted model 1^c^			Adjusted model 2^d^		
	PRR	95% CI	*p*	PRR	95% CI	*p*	PRR	95% CI	*p*
**Events of any grade**									
Aerobic capacity [per watt/kg bodyweight]	0.66	[0.59, 0.73]	<.001	0.71	[0.63, 0.81]	<.001	0.71	[0.63, 0.81]	<.001
Hand‐grip strength [per kg/kg bodyweight]	0.44	[0.29, 0.66]	<.001	0.83	[0.50, 1.39]	.485	0.60	[0.35, 1.03]	.063
Lower body endurance [per 5 repetitions]	0.94	[0.92, 0.96]	<.001	0.95	[0.93, 0.97]	<.001	0.95	[0.93, 0.97]	<.001
Composite fitness score^e^ [per *z* score]	0.79	[0.74, 0.85]	<.001	0.83	[0.77, 0.89]	<.001	0.81	[0.75, 0.88]	<.001
**Events grades 2+**									
Aerobic capacity	0.64	[0.56, 0.74]	<.001	0.69	[0.58, 0.81]	<.001	0.71	[0.59, 0.84]	<.001
Hand‐grip strength	0.55	[0.32, 0.96]	.036	1.23	[0.62, 2.45]	.557	0.86	[0.41, 1.879	.690
Lower body endurance	0.94	[0.91, 0.96]	<.001	0.94	[0.92, 0.97]	<.001	0.95	[0.92, 0.98]	.003
Composite fitness score	0.81	[0.74, 0.89]	<.001	0.85	[0.77, 0.94]	.002	0.84	[0.76, 0.93]	.001
**Events grades 3+**									
Aerobic capacity	0.55	[0.43, 0.70]	<.001	0.61	[0.46, 0.82]	.001	0.64	[0.47, 0.86]	.003
Hand‐grip strength	0.55	[0.32, 0.96]	.036	0.97	[0.30, 3.22]	.966	0.70	[0.19, 2.53]	.587
Lower body endurance	0.90	[0.85, 0.94]	<.001	0.91	[0.86, 0.96]	.001	0.92	[0.87, 0.98]	.002
Composite fitness score	0.73	[0.62, 0.85]	<.001	0.89	[0.68, 0.95]	.011	0.79	[0.66, 0.94]	.009

Abbreviations: BMI, body mass index; CI, confidence interval; PRR, prevalence rate ratio.

^a^ *n* = 159 for aerobic capacity, *n* = 160 for hand‐grip strength, *n* = 163 for lower body endurance, *n* = 163 for composite fitness score.

^b^ Basic model 0: adjusted for age, sex, and time since diagnosis.

^c^ Adjusted model 1: model 0 + BMI.

^d^ Adjusted model 2: model 1 + cancer types, anthracycline dose, steroid dose, location of radiotherapy. We calculated the cumulative anthracyclines dose as doxorubicin isotoxic equivalent dose (mg/m^2^) and the cumulative steroid dose as prednisone equivalent dose (mg/m^2^). Patients who did not receive steroid or anthracycline treatment, received 0 mg/m^2^ as their dose; six participants who received steroids but had missing information on cumulative dose were imputed with the median dose of all participants with steroid treatment.

^e^ Average *z* score of aerobic capacity, hand‐grip strength and lower body endurance.

When comparing the estimates using their *z* scores, strongest associations were seen for the composite fitness score, followed by aerobic capacity and lower body endurance (Table S3). The sensitivity analyses including only maximal work rate (Table S4) and adjusting fitness‐parameters for LBM (Table S5) were similar to the main results.

## DISCUSSION

### Main findings

Our study found a high burden of adverse health outcomes experienced by survivors years after childhood cancer diagnosis eligible for a fitness intervention and capable of completing CPET and strength testing, thus likely more healthy than the source population. Nearly all survivors reported at least one adverse health outcome, regardless of primary cancer diagnosis. Better physical fitness was associated with having fewer adverse health outcomes regardless of the survivor’s cancer history.

### Comparison with other studies

Our findings align with a US study reporting that ≥95% of 45‐year‐old CCS experience at least one adverse health outcome, 2.5–5.2 as much as their siblings.^6^ We found that 99% of survivors had already developed one adverse health outcome by a younger median age of 31 years. Hudson and colleagues^6^ further stated that 68% of survivors suffered from “serious/disabling or life‐threatening chronic conditions,” which was higher than 39% in our study, possibly due to the lower median age in our cohort. Lifestyle may also explain part of this difference; 34% of our cohort was overweight or obese compared to 66% in the US cohort.^52^ Less than half of the US survivors were physically active, whereas 84% of our cohort was physically active.^6^, ^34^, ^37^

Consistent with other studies, musculoskeletal disorders were the most common disorders, especially prominent in survivors of bone cancer. The Swiss Childhood Cancer Survivor Study reported a prevalence of musculoskeletal health conditions of 26% and a prevalence of 64% in bone‐tumor survivors.^53^ Musculoskeletal disorders have been linked to the cancer itself,^54^ and to cancer treatment directly (corticosteroids and antimetabolites inhibiting formation of new bone)^36^, ^55^ and indirectly (radiation induced hypothalamic‐pituitary‐axis dysfunction, hypogonadism, and growth hormone deficiency [GHD]) affecting the musculoskeletal system.^18^, ^36^, ^54^, ^56^ This may explain the dominance of musculoskeletal disorders in our study population; seven participants had GHD, 28 received cranial radiation (of which 18% showed GHD), and 16 had gonadal insufficiency. This is concerning, as physical limitations complicate everyday tasks and ultimately limit engagement in a physically active lifestyle.

Several studies assessed psychological adverse health outcomes with inconsistent results. Psychological conditions were the second most common events (49%) in our study. An American literature review, also based on self‐reported questionnaires, reports 11%–13% of survivors suffer from depression, lower than our 17%, and 6%–10% from anxiety, which aligns to our 9%.^57^ However, survivors of acute lymphoblastic leukemia reported similar or even higher health‐related quality of life^58^ and lower psychological distress than healthy peers.^16^ These discrepancies likely stem from varied (often self‐reported) outcome measures, and control group selection. Randomized controlled trials (RTC) show that aerobic exercise interventions can decrease depression among adults with cancer.^59^ Fit4life is the only RTC to assess physical activity’s mental health impact in pediatric survivors, although as a secondary outcome in leukemia survivors only.^23^ Further research on exercise interventions for mental health outcomes is necessary as exercise may offer a low‐risk therapeutic addition/alternative.

Overall, we found few differences between the tumor subgroups. Apart from nervous system and ear‐and‐labyrinth disorders (mostly impaired hearing), which were more prevalent in CNS‐tumor survivors, consistent with the neurotoxicity of platinum‐based chemotherapy used in pediatric CNS malignancies,^49^ no consistent pattern emerged in favor of or against any tumor type. Although severe adverse events varied slightly among the tumor groups, the overall low number of occurrences prevents us from drawing clinical conclusions.

### Interpretation of results

Cancer treatment is toxic and nearly all survivors reported adverse health outcomes. Better physical fitness was associated with reduced disease burden in every fitness parameter. This association remained regardless of the survivor’s cancer‐ or treatment type. Most likely, the association is bi‐directional; through cancer and its toxic treatment patients experience a loss of physical functioning and adverse health conditions,^55^, ^60^ furthering overall low fitness and a vicious cycle of further deconditioning and physical inactivity.^33^, ^61^, ^62^

Opposed to cancer treatments, fitness levels can be improved through physical activity intervention and may mitigate some of the health burdens of survivors.^14^, ^33^, ^34^ Aerobic fitness has been shown to be one of the strongest predictors of cardiovascular morbidity in population‐based studies and in cancer patients.^37^, ^63^ Treatment‐related risk factors are difficult to modify,^60^ however, cardiovascular fitness can be improved through lifestyle interventions and positively impact cardiovascular disease in CCS.^37^ Higher muscle strength is a predictor for reduced cardiovascular disease risk,^19^, ^37^ less fatigue,^33^, ^62^ and longer survival^64^, ^65^, ^66^ of cancer survivors.

Musculoskeletal disorders are concerning adverse health outcomes because they increase the risk of fractures and immobility, and therefore impact the individual and socioeconomic burden. Poor bone health promotes physical decompensation and reduces physical fitness, which in turn negatively affects bone health. Simple exercise interventions are feasible and could help break this cycle.^36^ Exercise interventions in CCS showed that improved physical fitness leads to reduced cardiovascular disease risk,^22^, ^23^, ^67^, ^68^, ^69^, ^70^ with higher physical activity leading to greater risk‐reduction,^34^ better bone health,^38^, ^71^, ^72^ less fatigue,^33^, ^61^, ^73^ psychological stability,^33^, ^74^ and better quality of life.^33^

### Clinical implications

Our study showed that physical fitness is associated with fewer adverse health outcomes regardless of cancer type and treatment history. This relation was found in a selective population of CCS, which was partly already physically active, and therefore possibly healthier. This may have lowered the strength of associations by limiting our sample to the more fit and less sick CCS. However, it remains a challenge to reach behavioral changes,^35^, ^75^ especially considering the high prevalence of psychological conditions and fatigue interfering with exercise motivation and implementation of lifestyle changes. Because the focus during follow‐up care is on somatic illnesses, possible psychiatric conditions could be overlooked.^57^, ^76^, ^77^, ^78^ Clinicians should address psychiatric symptoms and fatigue and involve experienced specialists including psycho‐oncologists to guide PA implementation when needed.

Causality between physical fitness and adverse health outcomes has been shown in other diseases,^79^ but needs to be proven after childhood cancer. Nonetheless, a simple clinical examination as the STS could be easily integrated in clinical practice to assess patients` fitness and guide clinicians in lifestyle education of survivors.

### Strengths and limitations

This study included a comprehensive and standardized assessment of a wide range of possible adverse health outcomes in survivors of childhood cancer.^41^ This study is among the first to assess three important physical fitness components in CCS and relate them to adverse health outcomes.

Findings should be interpreted in the context of study limitations. The absence of a control group prevents us from determining the attributable fraction of adverse health outcomes related to childhood cancer and/or its treatment. The cross‐sectional design limits our ability to establish causality between health outcomes and physical fitness; the adverse health outcomes happened between cancer diagnosis and study visit, whereas physical fitness was assessed at study start. However, we view physical fitness as a long‐term construct shaped by lifestyle. The observed associations may be influenced by unmeasured confounders (genetic predisposition) or mediators (socioeconomic and lifestyle factors) that were not adjusted for.

Comprehensive clinical assessments and aim of a physical activity trial may have reduced participation rates leading to a selective fitter and likely healthier population, which may explain the absence of differences by tumor type.^80^ Including a more general population of CCS (e.g., less physically active/fit) may make our association of physical fitness to adverse health outcomes even stronger.

Assessment of fitness parameters showed some limitations. First, there was an overlap between predictor and outcome variables, as reduced fitness performance, used as a proxy for musculoskeletal weakness, was also classified as a CTCAE event. Second, because CPET primarily assesses aerobic capacity, its inclusion to “generalized muscle weakness” may have led to an over reporting of musculoskeletal disorders. Third, because submaximal CPET test were included in the analysis, it cannot be distinguished if reduced watt/kg was reached due to the inability to reach maximal performance versus lacking motivation. Nevertheless, sensitivity analyses including only those with maximal tests did not reveal different results.

Our DXA‐derived bone mineral density outcomes were not adjusted for body size, causing potential over reporting of osteoporosis due to short stature.^81^

Cumulative dose was only available for anthracyclines and steroids due to the primary objective of the SURfit study, and not for other therapies, including alkylating agents, possibly also contributing to the burden of chronic health conditions.

Self‐reported depressive or anxiety symptoms by BSI were equated to grade 1 events, which may have led to over reporting of mental health outcomes. Finally, time from primary cancer diagnosis to adverse event was not available, as most event onset dates were not recorded. As a result, person‐years could not be calculated.

In conclusion, this study emphasizes the substantial burden of adverse health outcomes among young adult CCS that may be underestimated due to the selective population of already physically active and possible healthier CCS. Improved physical fitness is a modifiable factor that might mitigate these adverse events for all CCS, regardless of their cancer history and activity level, however, future research is needed to ascertain causality and its strength of association. Simple physical fitness tests may be integrated into the regular care of CCS to identify survivors potentially at risk for higher morbidity and guide them on how to improve their physical fitness.

## AUTHOR CONTRIBUTIONS

**Anna K. Mayr**: Software, data curation, validation, formal analysis, visualization, writing–original draft, and writing–review and editing. **Simeon Zürcher**: Investigation, project administration, and writing–review and editing. **Iris Bänteli**: Investigation, project administration, and writing–review and editing. **Helge Hebestreit**: Writing–review and editing. **Rahel Kasteler**: Investigation and writing–review and editing. **Nicolas X. von der Weid**: Investigation, funding acquisition, project administration, resources, and writing–review and editing. **Susi Kriemler**: Conceptualization, methodology, investigation, validation, supervision, funding acquisition, visualization, project administration, resources, and writing–review and editing. **Christina Schindera**: Conceptualization, methodology, data curation, investigation, validation, supervision, funding acquisition, visualization, project administration, resources, and writing–review and editing. **Corina S. Rueegg**: Conceptualization, methodology, software, data curation, validation, supervision, funding acquisition, visualization, project administration, and writing–review and editing.

## CONFLICT OF INTEREST STATEMENT

The authors declare no conflicts of interest.

## Supporting information

Supplementary Material

Supplementary Material

## Data Availability

De‐identified individual participant data that underlie the results reported in this article, statistical programs and data dictionary are available on request to the corresponding author, immediately following publication and without end date, to anyone who provides a sound proposal. The study protocol, statistical analysis plan, patient information, and informed consent forms of the SURfit study are published at the Open Science Framework platform: https://osf.io/w6j4y/.

## References

[1] Curry HL , Parkes SE , Powell JE , Mann JR . Caring for survivors of childhood cancers: the size of the problem. Eur J Cancer. 2006;42(4):501‐508. doi:10.1016/j.ejca.2005.11.003 16406574

[2] American Cancer Society . Key Statistics for Childhood Cancers. American Cancer Society; 2024. Accessed January 9, 2025. https://www.cancer.org/cancer/types/cancer‐in‐children/key‐statistics.html#:~:text=at%20least%201975.‐,Survival%20rates%20for%20children%20with%20cancer,survival%20rate%20was%20about%2058%25

[3] Gatta G , Zigon G , Capocaccia R , et al. Survival of European children and young adults with cancer diagnosed 1995‐2002. Eur J Cancer. 2009;45(6):992‐1005. doi:10.1016/j.ejca.2008.11.042 19231160

[4] Cardous‐Ubbink MC , Heinen RC , Langeveld NE , et al. Long‐term cause‐specific mortality among five‐year survivors of childhood cancer. Pediatr Blood Cancer. 2004;42(7):563‐573. doi:10.1002/pbc.20028 15127410

[5] Esbenshade AJ , Lu L , Friedman DL , et al. Accumulation of chronic disease among survivors of childhood cancer predicts early mortality. J Clin Oncol. 2023;41(20):3629‐3641. doi:10.1200/jco.22.02240 37216619 PMC10325751

[6] Hudson MM , Ness KK , Gurney JG , et al. Clinical ascertainment of health outcomes among adults treated for childhood cancer. JAMA. 2013;309(22):2371‐2381. doi:10.1001/jama.2013.6296 23757085 PMC3771083

[7] Eton DT , Anderson RT , Cohn WF , et al. Risk factors for poor health‐related quality of life in cancer survivors with multiple chronic conditions: exploring the role of treatment burden as a mediator. Patient Relat Outcome Meas. 2019;10:89‐99. doi:10.2147/prom.s191480 30962731 PMC6432889

[8] Corbett T , Bridges J . Multimorbidity in older adults living with and beyond cancer. Curr Opin Support Palliat Care. 2019;13(3):220‐224. doi:10.1097/spc.0000000000000439 31157655

[9] Harrington RL , Qato DM , Antoon JW , Caskey RN , Schumock GT , Lee TA . Impact of multimorbidity subgroups on the health care use of early pediatric cancer survivors. Cancer. 2020;126(3):649‐658. doi:10.1002/cncr.32201 31639197

[10] Miser JS , Shia BC , Kao YW , Liu YL , Chen SY , Ho WL . The health care utilization and medical costs in long‐term follow‐up of children diagnosed with leukemia, solid tumor, or brain tumor: population‐based study using the National Health Insurance claims data. JMIR Public Health Surveill. 2023;9:e42350. doi:10.2196/42350 36862495 PMC10020904

[11] Lee IM , Shiroma EJ , Lobelo F , et al. Effect of physical inactivity on major non‐communicable diseases worldwide: an analysis of burden of disease and life expectancy. Lancet. 2012;380(9838):219‐229. doi:10.1016/s0140-6736(12)61031-9 22818936 PMC3645500

[12] Morishita S , Suzuki K , Okayama T , et al. Recent findings in physical exercise for cancer survivors. Phys Ther Res. 2023;26(1):10‐16. doi:10.1298/ptr.r0023 37181484 PMC10169310

[13] Campbell KL , Winters‐Stone KM , Wiskemann J , et al. Exercise guidelines for cancer survivors: consensus statement from international multidisciplinary roundtable. Med Sci Sports Exerc. 2019;51(11):2375‐2390. doi:10.1249/mss.0000000000002116 31626055 PMC8576825

[14] Irestorm E , van Gorp M , Twisk J , et al. Longitudinal development of fatigue after treatment for childhood cancer: a national cohort study. Acta Oncol. 2023;62(10):1309‐1321. doi:10.1080/0284186x.2023.2254477 37676687

[15] Mack JW . Exercise and well‐being in adult survivors of childhood cancer‐time for interventions. JAMA Oncol. 2020;6(8):1170‐1171. doi:10.1001/jamaoncol.2020.1658 32584380

[16] Michel G , Rebholz CE , von der Weid NX , Bergstraesser E , Kuehni CE . Psychological distress in adult survivors of childhood cancer: the Swiss Childhood Cancer Survivor study. J Clin Oncol. 2010;28(10):1740‐1748. doi:10.1200/jco.2009.23.4534 20194864

[17] Armand A , Rochette E , Grèze V , et al. Fitness and metabolic response to exercise in young adult survivors of childhood lymphoma. Support Care Cancer. 2023;31(6):358. doi:10.1007/s00520-023-07812-5 37247034

[18] Guida JL , Hyun G , Belsky DW , et al. Associations of seven measures of biological age acceleration with frailty and all‐cause mortality among adult survivors of childhood cancer in the St. Jude Lifetime Cohort. Nat Cancer. 2024;5(5):731‐741. doi:10.1038/s43018-024-00745-w 38553617 PMC11139608

[19] Wogksch MD , Goodenough CG , Finch ER , Partin RE , Ness KK . Physical activity and fitness in childhood cancer survivors: a scoping review. Aging Cancer. 2021;2(4):112‐128. doi:10.1002/aac2.12042 35098147 PMC8794406

[20] Armstrong GT , Oeffinger KC , Chen Y , et al. Modifiable risk factors and major cardiac events among adult survivors of childhood cancer. J Clin Oncol. 2013;31(29):3673‐3680. doi:10.1200/jco.2013.49.3205 24002505 PMC3804290

[21] Bekhet AH , Abdallah AR , Ismail HM , et al. Benefits of aerobic exercise for breast cancer survivors: a systematic review of randomized controlled trials. Asian Pac J Cancer Prev. 2019;20(11):3197‐3209. doi:10.31557/apjcp.2019.20.11.3197 31759342 PMC7063018

[22] Blaauwbroek R , Bouma MJ , Tuinier W , et al. The effect of exercise counselling with feedback from a pedometer on fatigue in adult survivors of childhood cancer: a pilot study. Support Care Cancer. 2009;17(8):1041‐1048. doi:10.1007/s00520-008-0533-y 19015892 PMC2707951

[23] Huang JS , Dillon L , Terrones L , et al. Fit4Life: a weight loss intervention for children who have survived childhood leukemia. Pediatr Blood Cancer. 2014;61(5):894‐900. doi:10.1002/pbc.24937 24436138 PMC3997743

[24] Gatta G , Capocaccia R , Coleman MP , Ries LA , Berrino F . Childhood cancer survival in Europe and the United States. Cancer. 2002;95(8):1767‐1772. doi:10.1002/cncr.10833 12365026

[25] Chen JZ , Liang B . Comparison of American and European guidelines for cardio‐oncology of heart failure. Heart Fail Rev. 2023;28(5):1211‐1220. doi:10.1007/s10741-023-10304-7 36912998

[26] Wheaton S . Why you’re more likely to die of cancer in Europe than America Brussels. Politico 2019. 2024. Accessed October 28, 2024. https://www.politico.eu/article/cancer‐europe‐america‐comparison/

[27] Babecoff S , Mermillod F , Marino D , et al. Long‐term follow‐up for childhood cancer survivors: the Geneva experience. Swiss Med Wkly. 2022;152(1314):w30153. doi:10.4414/smw.2022.w30153 35429234

[28] Byrne J , Schmidtmann I , Rashid H , et al. Impact of era of diagnosis on cause‐specific late mortality among 77 423 five‐year European survivors of childhood and adolescent cancer: the PanCareSurFup consortium. Int J Cancer. 2022;150(3):406‐419. doi:10.1002/ijc.33817 34551126

[29] Garwicz S , Anderson H , Olsen JH , et al. Late and very late mortality in 5‐year survivors of childhood cancer: changing pattern over four decades‐‐experience from the Nordic countries. Int J Cancer. 2012;131(7):1659‐1666. doi:10.1002/ijc.27393 22170520

[30] Teepen JC , Kok JL , Feijen EAM , et al. Questionnaire‐ and linkage‐based outcomes in Dutch childhood cancer survivors: methodology of the DCCSS LATER study part 1. Cancer Med. 2023;12(6):7588‐7602. doi:10.1002/cam4.5519 36519590 PMC10067029

[31] Streefkerk N , Teepen JC , Feijen EAM , et al. The cumulative burden of self‐reported, clinically relevant outcomes in long‐term childhood cancer survivors and implications for survivorship care: A DCCSS LATER study. Cancer. 2024;130(8):1349‐1358. doi:10.1002/cncr.35148 38100618

[32] Asdahl PH , Winther JF , Bonnesen TG , et al. The Adult Life After Childhood Cancer in Scandinavia (ALiCCS) study: design and characteristics. Pediatr Blood Cancer. 2015;62(12):2204‐2210. doi:10.1002/pbc.25661 26193842

[33] Deng WH , Zürcher SJ , Schindera C , et al. Effect of a 1‐year physical activity intervention on quality of life, fatigue, and distress in adult childhood cancer survivors—a randomized controlled trial (SURfit). Cancer. 2024;130(10):1869‐1883. doi:10.1002/cncr.35207 38315522

[34] Rueegg CS , Zürcher SJ , Schindera C , et al. Effect of a 1‐year physical activity intervention on cardiovascular health in long‐term childhood cancer survivors‐a randomised controlled trial (SURfit). Br J Cancer. 2023;129(8):1284‐1297. doi:10.1038/s41416-023-02410-y 37653075 PMC10575964

[35] Jung R , Zürcher SJ , Schindera C , et al. Adherence and contamination in a 1‐year physical activity program in childhood cancer survivors: a report from the SURfit study. Cancer Med. 2023;12(13):14731‐14741. doi:10.1002/cam4.6096 37199378 PMC10358195

[36] Jung R , Zürcher SJ , Schindera C , et al. Effect of a physical activity intervention on lower body bone health in childhood cancer survivors: a randomized controlled trial (SURfit). Int J Cancer. 2023;152(2):162‐171. doi:10.1002/ijc.34234 35913755 PMC9805122

[37] Schindera C , Zürcher SJ , Jung R , et al. Physical fitness and modifiable cardiovascular disease risk factors in survivors of childhood cancer: a report from the SURfit study. Cancer. 2021;127(10):1690‐1698. doi:10.1002/cncr.33351 33405260

[38] Zürcher SJ , Jung R , Monnerat S , et al. High impact physical activity and bone health of lower extremities in childhood cancer survivors: a cross‐sectional study of SURfit. Int J Cancer. 2020;147(7):1845‐1854. doi:10.1002/ijc.32963 32167159

[39] Rueegg CS , Kriemler S , Zuercher SJ , et al. A partially supervised physical activity program for adult and adolescent survivors of childhood cancer (SURfit): study design of a randomized controlled trial [NCT02730767]. BMC Cancer. 2017;17(1):822. doi:10.1186/s12885-017-3801-8 29207962 PMC5717834

[40] National Cancer Institute . Surveillance, Epidemiology and End Results Program. 2017. Accessed April 3, 2024. https://seer.cancer.gov/iccc/

[41] National Cancer Institute . Cancer Therapy Evaluation Program (CTEP). US Department of Health and Human Services; 2021. Accessed April 3, 2024. https://ctep.cancer.gov/

[42] Derogatis LR , Melisaratos N . The brief symptom inventory: an introductory report. Psychol Med. 1983;13(3):595‐605. doi:10.1017/s0033291700048017 6622612

[43] Vercoulen JH , Swanink CM , Fennis JF , Galama JM , van der Meer JW , Bleijenberg G . Dimensional assessment of chronic fatigue syndrome. J Psychosom Res. 1994;38(5):383‐392. doi:10.1016/0022-3999(94)90099-x 7965927

[44] Godfrey S . Exercise tests in assessing children with lung or heart disease. Thorax. 1970;25(2):258. doi:10.1136/thx.25.2.258 PMC472174 5442010

[45] Werle S , Goldhahn J , Drerup S , Simmen BR , Sprott H , Herren DB . Age‐ and gender‐specific normative data of grip and pinch strength in a healthy adult Swiss population. J Hand Surg Eur Vol. 2009;34(1):76‐84. doi:10.1177/1753193408096763 19129352

[46] Radtke T , Puhan MA , Hebestreit H , Kriemler S . The 1‐min sit‐to‐stand test‐‐A simple functional capacity test in cystic fibrosis? J Cyst Fibros. 2016;15(2):223‐226. doi:10.1016/j.jcf.2015.08.006 26363563

[47] Vande Poppe DJ , Hulzebos E , Takken T , Group L‐LFRS . Reference values for maximum work rate in apparently healthy Dutch/Flemish adults: data from the LowLands fitness registry. Acta Cardiol. 2019;74(3):223‐230. doi:10.1080/00015385.2018.1478763 29933724

[48] Strassmann A , Steurer‐Stey C , Lana KD , et al. Population‐based reference values for the 1‐min sit‐to‐stand test. Int J Publ Health. 2013;58(6):949‐953. doi:10.1007/s00038-013-0504-z 23974352

[49] Children's Oncology Group . Long‐Term Follow‐Up Guidelines for Survivors of Childhood, Adolescent, and Young Adult Cancers. 6. Accessed November 2, 2024. http://www.survivorshipguidelines.org/ 10.1001/jamaoncol.2024.6812 PMC12188901 39976936

[50] Inaba H , Pui CH . Glucocorticoid use in acute lymphoblastic leukaemia. Lancet Oncol. 2010;11(11):1096‐1106. doi:10.1016/s1470-2045(10)70114-5 20947430 PMC3309707

[51] World Health Organization. Everyday actions for better health – WHO recommendations. Accessed October 25, 2024. https://www.who.int/europe/news‐room/fact‐sheets/item/a‐healthy‐lifestyle‐‐‐who‐recommendations

[52] Smith WA , Li C , Nottage KA , et al. Lifestyle and metabolic syndrome in adult survivors of childhood cancer: a report from the St. Jude Lifetime Cohort Study. Cancer. 2014;120(17):2742‐2750. doi:10.1002/cncr.28670 25070001 PMC4165406

[53] Christen S , Roser K , Mader L , et al. Incidence and prevalence of musculoskeletal health conditions in survivors of childhood and adolescent cancers: a report from the Swiss Childhood Cancer Survivor Study. Cancer Med. 2024;13(8):e7204. doi:10.1002/cam4.7204 38650581 PMC11036073

[54] Wilson CL , Ness KK . Bone mineral density deficits and fractures in survivors of childhood cancer. Curr Osteoporos Rep. 2013;11(4):329‐337. doi:10.1007/s11914-013-0165-0 24043370 PMC4260527

[55] Brown SA , Guise TA . Cancer treatment‐related bone disease. Crit Rev Eukaryot Gene Expr. 2009;19(1):47‐60. doi:10.1615/critreveukargeneexpr.v19.i1.20 19191756 PMC2762110

[56] Wasilewski‐Masker K , Kaste SC , Hudson MM , Esiashvili N , Mattano LA , Meacham LR . Bone mineral density deficits in survivors of childhood cancer: long‐term follow‐up guidelines and review of the literature. Pediatrics. 2008;121(3):e705‐e713. doi:10.1542/peds.2007-1396 18310191

[57] Zeltzer L , Recklitis C , Buchbinder D , et al. Psychological status in childhood cancer survivors: a report from the Childhood Cancer Survivor Study. J Clin Oncol. 2010;27(27):2396‐2404. doi:10.1200/jco.2008.21.1433 PMC2677925 19255309

[58] Essig S , von der Weid NX , Strippoli MP , et al. Health‐related quality of life in long‐term survivors of relapsed childhood acute lymphoblastic leukemia. PLoS One. 2012;7(5):e38015. doi:10.1371/journal.pone.0038015 22662262 PMC3360640

[59] Kulchycki M , Halder HR , Askin N , et al. Aerobic physical activity and depression among patients with cancer: a systematic review and meta‐analysis. JAMA Netw Open. 2024;7(10):e2437964. doi:10.1001/jamanetworkopen.2024.37964 39378035 PMC11581595

[60] Armstrong GT , Ross JD . Late cardiotoxicity in aging adult survivors of childhood cancer. Prog Pediatr Cardiol. 2014;36(1‐2):19‐26. doi:10.1016/j.ppedcard.2014.09.003 26412958 PMC4580976

[61] Ernst M , Wagner C , Oeser A , et al. Resistance training for fatigue in people with cancer. Cochrane Database Syst Rev. 2024;11(11):CD015518. doi:10.1002/14651858.CD015518 39606939 PMC11603558

[62] Dimeo F , Stieglitz RD , Novelli‐Fischer U , Fetscher S , Mertelsmann R , Keul J . Correlation between physical performance and fatigue in cancer patients. Ann Oncol. 1997;8(12):1251‐1255. doi:10.1023/a:1008234310474 9496391

[63] Kodama S , Saito K , Tanaka S , et al. Cardiorespiratory fitness as a quantitative predictor of all‐cause mortality and cardiovascular events in healthy men and women: a meta‐analysis. JAMA. 2009;301(19):2024‐2035. doi:10.1001/jama.2009.681 19454641

[64] Kilgour RD , Vigano A , Trutschnigg B , Lucar E , Borod M , Morais JA . Handgrip strength predicts survival and is associated with markers of clinical and functional outcomes in advanced cancer patients. Support Care Cancer. 2013;21(12):3261‐3270. doi:10.1007/s00520-013-1894-4 23872952

[65] Versteeg KS , Blauwhoff‐Buskermolen S , Buffart LM , et al. Higher muscle strength is associated with prolonged survival in older patients with advanced cancer. Oncologist. 2018;23(5):580‐585. doi:10.1634/theoncologist.2017-0193 29222198 PMC5947445

[66] Segal R , Zwaal C , Green E , et al. Exercise for people with cancer: a clinical practice guideline. Curr Oncol. 2017;24(1):40‐46. doi:10.3747/co.24.3376 28270724 PMC5330628

[67] Li WHC , Ho KY , Lam KKW , et al. Adventure‐based training to promote physical activity and reduce fatigue among childhood cancer survivors: a randomized controlled trial. Int J Nurs Stud. 2018;83:65‐74. doi:10.1016/j.ijnurstu.2018.04.007 29689482

[68] Ruble K , Scarvalone S , Gallicchio L , Davis C , Wells D . Group physical activity intervention for childhood cancer survivors: a pilot study. J Phys Act Health. 2016;13(3):352‐359. doi:10.1123/jpah.2015-0050 26284383

[69] Howell CR , Krull KR , Partin RE , et al. Randomized web‐based physical activity intervention in adolescent survivors of childhood cancer. Pediatr Blood Cancer. 2018;65(8):e27216. doi:10.1002/pbc.27216 29722481 PMC6019155

[70] Sabel M , Sjölund A , Broeren J , et al. Active video gaming improves body coordination in survivors of childhood brain tumours. Disabil Rehabil. 2016;38(21):2073‐2084. doi:10.3109/09638288.2015.1116619 26728453

[71] Gunter KB , Almstedt HC , Janz KF . Physical activity in childhood may be the key to optimizing lifespan skeletal health. Exerc Sport Sci Rev. 2012;40(1):13‐21. doi:10.1097/jes.0b013e318236e5ee 21918458 PMC3245809

[72] Bauer JJ , Snow CM . What is the prescription for healthy bones? J Musculoskelet Neuronal Interact. 2003;3(4):352‐356. 15758321

[73] Hawn R , Stevens J , Basha M , Kwekkeboom K . A systematic review of the characteristics and effects of physical activity interventions on physical activity engagement, long‐term and late effects, and quality of life in adolescent and young adult cancer survivors. J Adolesc Young Adult Oncol. 2024;13(3):444‐464. doi:10.1089/jayao.2023.0150 38324011

[74] Moraitis AM , Seven M , Walker RK . Physical activity in young adult cancer survivors: a scoping review. Oncol Nurs Forum. 2021;48(2):184‐194. doi:10.1188/21.onf.184-194 33600391

[75] Salisbury CE , Hyde MK , Cooper ET , Stennett RC , Gomersall SR , Skinner TL . Physical activity behaviour change in people living with and beyond cancer following an exercise intervention: a systematic review. J Cancer Surviv. 2023;17(3):569‐594. doi:10.1007/s11764-023-01377-2 37074621 PMC10209249

[76] Götze H , Friedrich M , Taubenheim S , Dietz A , Lordick F , Mehnert A . Depression and anxiety in long‐term survivors 5 and 10 years after cancer diagnosis. Support Care Cancer. 2020;28(1):211‐220. doi:10.1007/s00520-019-04805-1 31001695

[77] Walker J , Hansen CH , Martin P , et al. Prevalence, associations, and adequacy of treatment of major depression in patients with cancer: a cross‐sectional analysis of routinely collected clinical data. Lancet Psychiatry. 2014;1(5):343‐350. doi:10.1016/s2215-0366(14)70313-x 26360998

[78] Fernando A , Tokell M , Ishak Y , Love J , Klammer M , Koh M . Mental health needs in cancer ‐ a call for change. Future Healthc J. 2023;10(2):112‐116. doi:10.7861/fhj.2023-0059 PMC10540791 37786642

[79] Blair SN , Kohl HW , Paffenbarger RS , Clark DG , Cooper KH , Gibbons LW . Physical fitness and all‐cause mortality. A prospective study of healthy men and women. JAMA. 1989;262(17):2395‐2401. doi:10.1001/jama.1989.03430170057028 2795824

[80] Lesser IA , Wurz A , Bean C , Culos‐Reed N , Lear SA , Jung M . Participant bias in community‐based physical activity research: a consistent limitation? J Phys Act Health. 2024;21(2):109‐112. doi:10.1123/jpah.2023-0267 37935192

[81] Zemel BS , Leonard MB , Kelly A , et al. Height adjustment in assessing dual energy x‐ray absorptiometry measurements of bone mass and density in children. J Clin Endocrinol Metab. 2010;95(3):1265‐1273. doi:10.1210/jc.2009-2057 20103654 PMC2841534

