# Supplementary material for: Physical fitness and clinically assessed disease burden in long‐term childhood cancer survivors—The SURfit study
Source: Cancer. 2025 Aug 19;131(17):e70051. doi: 10.1002/cncr.70051 (PMC12365372; doi:10.1002/cncr.70051)
Supplement: Supplementary file 1 — Supplementary Material [file CNCR-131-e70051-s002.docx]

Supplemental Figures


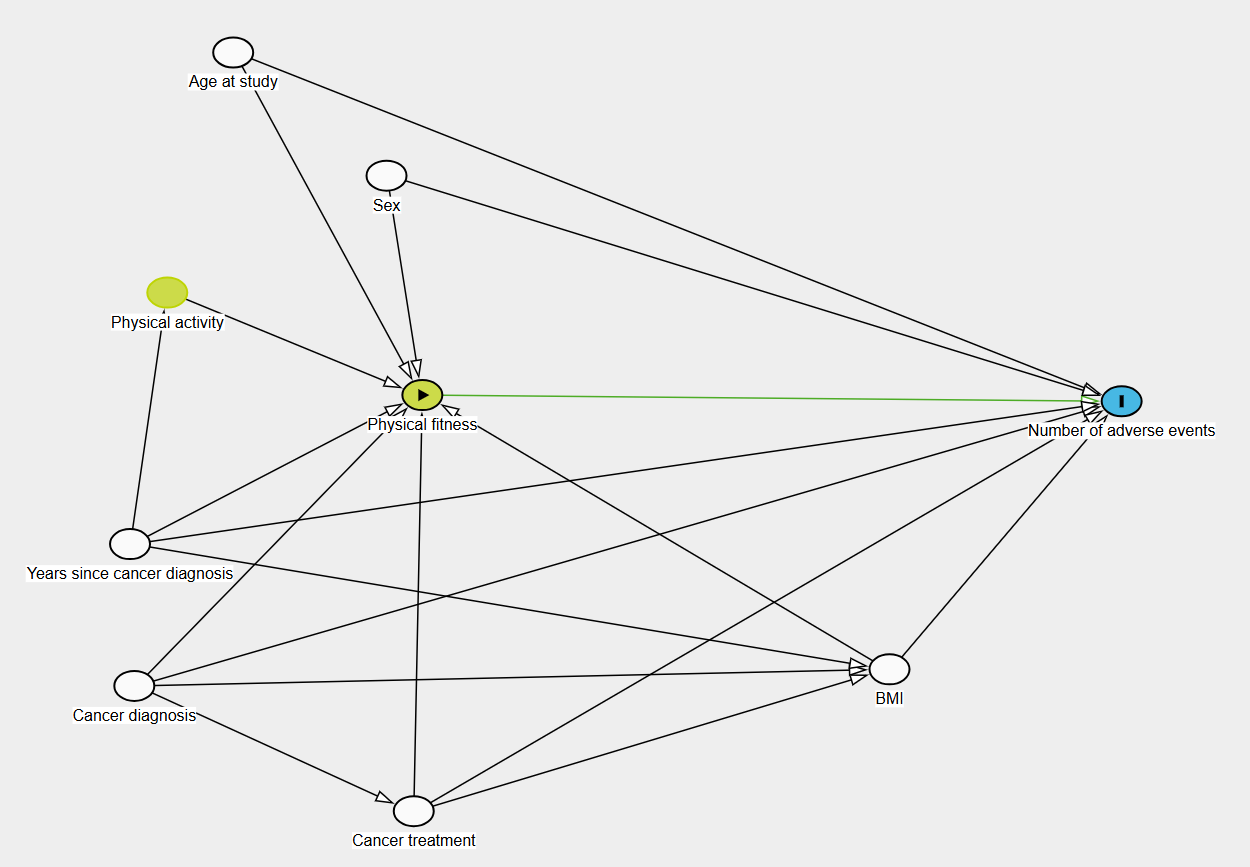
 **Supplemental Figure 1. Directed acyclic graph of mediators and covariates for the causal relationship between physical fitness and number of adverse events.** The directed acyclic graph (DAG) explains the causal assumptions for the effect of physical fitness (aerobic capacity, hand-grip strength and lower body endurance) on the number of adverse events. Variables are displayed by color: Blue with black frame represents the outcome variable, green with black frame the exposure variable, green the ancestor of exposure and white with black frame the adjusted variable. The green arrow from physical fitness to number of adverse effects represents the assumed total effect. Black arrows represent covariates associated with physical fitness as well as the number of adverse events. Based on this DAG, the minimal adjustment set to calculate the total “effect” of physical fitness on number of adverse events are: sex, age at study, cancer diagnosis, years since cancer diagnosis, cancer treatment and BMI. Cancer treatment includes radiotherapy location (cranial, abdominal, total body irradiation, radiation of “other location” and “no radiotherapy”), cumulative anthracycline dose (mg/m^2^), and cumulative steroid dose (mg/m^2^).

Abbreviations: DAG, directed acyclic graph; BMI, body mass index.

**Supplemental Figure 2. Average number of adverse events per person for any grade (1-4), grades 2+ (2-4) and grades 3+ (3-4).** Figure 1 shows the average number of adverse events per person (diamonds) for different grades, according to the Common Terminology Criteria for Adverse Events (CTCAE). Results are presented overall (total) and stratified by primary cancer diagnosis. Error bars represent 95% Poisson distributed exact confidence intervals.

Abbreviations: CNS, central nervous system; CTCAE, common terminology criteria for adverse events.

**Supplemental Figure 3. Percent of childhood cancer survivors (CCS) with at least one event in the respective system organ class (SOC), categorized into grade 1, grade 2 and grades 3+ (3-4), stratified by primary cancer diagnosis.** (A) Leukemia, (B) Lymphoma, (C) CNS tumor, (D) Bone tumor and soft-tissue sarcoma, (E) Other tumor diagnoses. The full official name of each SOC is given in Supplemental Table 1. The SOCs are ordered alphabetically from top to bottom. The severity of event (grade) is represented by gray shading.

Abbreviations: CCS, childhood cancer survivors; CNS, central nervous system; SOC, system organ class.

Supplemental Tables

**Supplemental Table 1. Number of reported adverse events any grade (1-4) and** **grades 2+ (2-4) and 3+ (3-4), by System Organ Class (SOC) according to the Common Terminology Criteria for Adverse Events (CTCAE), *N=18-163***

|  |  | | | | **Total (N=163)** | | | | |  | | | | | |
| --- | --- | --- | --- | --- | --- | --- | --- | --- | --- | --- | --- | --- | --- | --- | --- |
|  | Events any grade (1-4) | | | | Events grades 2+ (2-4) | | | | | Events grades 3+ (3-4) | | | | | |
| **System Organ Class (SOC)** | N events^a^ | | N persons^b^ | (%)^c^ | N events^a^ | N persons^b^ | | (%)^c^ | | N events^a^ | | N persons^b^ | | (%)^c^ |  |
| Blood and lymphatic system disorders | 2 | 2 | | (1.2) | 0 | 0 | | (0.0) | | 0 | | 0 | | (0.0) |  |
| Cardiac disorders | 43 | 35 | | (21.5) | 11 | 11 | | (6.7) | | 3 | | 3 | | (1.8) |  |
| Congenital, familial and genetic disorders | 4 | 4 | | (2.5) | 3 | 3 | | (1.8) | | 2 | | 2 | | (1.2) |  |
| Ear and labyrinth disorders | 19 | 17 | | (10.4) | 7 | 6 | | (3.7) | | 4 | | 3 | | (1.8) |  |
| Endocrine disorders | 59 | 40 | | (24.5) | 46 | 32 | | (19.6) | | 6 | | 5 | | (3.1) |  |
| Eye disorders | 21 | 19 | | (11.7) | 19 | 17 | | (10.4) | | 8 | | 7 | | (4.3) |  |
| Gastrointestinal disorders | 30 | 26 | | (16.0) | 20 | 19 | | (11.7) | | 9 | | 8 | | (4.9) |  |
| General symptoms: pain, fatigue, edema | 69 | 60 | | (36.8) | 28 | 27 | | (16.6) | | 2 | | 2 | | (1.2) |  |
| Infections and infestations | 22 | 20 | | (12.3) | 20 | 17 | | (10.4) | | 19 | | 17 | | (10.4) |  |
| Injury, poisoning and procedural complications | 63 | 51 | | (31.3) | 57 | 47 | | (28.8) | | 29 | | 25 | | (15.3) |  |
| Laboratory, imaging and functional findings | 46 | 39 | | (23.9) | 16 | 15 | | (9.2) | | 2 | | 2 | | (1.2) |  |
| Metabolism and nutrition disorders | 74 | 65 | | (39.9) | 55 | 52 | | (31.9) | | 19 | | 18 | | (11.0) |  |
| Musculoskeletal and connective tissue disorders | 260 | 130 | | (79.8) | 100 | 69 | | (42.3) | | 14 | | 14 | | (8.6) |  |
| Neoplasms benign, malignant and unspecified (incl cysts and polyps) | 38 | 26 | | (16.0) | 26 | 20 | | (12.3) | | 19 | | 14 | | (8.6) |  |
| Nervous system disorders | 100 | 66 | | (40.5) | 41 | 32 | | (19.6) | | 14 | | 14 | | (8.6) |  |
| Pregnancy, puerperium and perinatal conditions | 5 | 4 | | (2.5) | 5 | 4 | | (2.5) | | 5 | | 4 | | (2.5) |  |
| Psychiatric disorders | 122 | 80 | | (49.1) | 35 | 28 | | (17.2) | | 6 | | 6 | | (3.7) |  |
| Renal and urinary disorders | 7 | 6 | | (3.7) | 6 | 5 | | (3.1) | | 5 | | 4 | | (2.5) |  |
| Reproductive system and breast disorders | 22 | 20 | | (12.3) | 17 | 16 | | (9.8) | | 3 | | 3 | | (1.8) |  |
| Respiratory, thoracic and mediastinal disorders | 45 | 36 | | (22.1) | 23 | 23 | | (14.1) | | 10 | | 10 | | (6.1) |  |
| Skin and subcutaneous tissue disorders | 13 | 12 | | (7.4) | 6 | 6 | | (3.7) | | 0 | | 0 | | (0.0) |  |
| Surgical and medical procedures | 66 | 53 | | (32.5) | 59 | 47 | | (28.8) | | 32 | | 24 | | (14.7) |  |
| Vascular disorders | 40 | 36 | | (22.1) | 36 | 33 | | (20.2) | | 8 | | 8 | | (4.9) |  |
| *All SOCs combined* | *1170* | *162* | | *(99.4)* | *636* | *153* | | *(93.9)* | | *220* | | *63* | | *(38.7)* |  |
|  | **Leukemia (N=57)** | | | | | | | | | | | | | | |
|  | Events any grade (1-4) | | | | Events grades 2+ (2-4) | | | | | Events grades 3+ (3-4) | | | | | |
| **System Organ Class** | N events^a^ | | N persons^b^ | (%)^c^ | N events^a^ | | N persons^b^ | | (%)^c^ | N events^a^ | N persons^b^ | | (%)^c^ | | |
| Blood and lymphatic system disorders | 1 | | 1 | (1.8) | 0 | | 0 | | (0.0) | 0 | | 0 | (0.0) | | |
| Cardiac disorders | 14 | | 10 | (17.5) | 2 | | 2 | | (3.5) | 0 | | 0 | (0.0) | | |
| Congenital, familial and genetic disorders | 1 | | 1 | (1.8) | 1 | | 1 | | (1.8) | 0 | | 0 | (0.0) | | |
| Ear and labyrinth disorders | 6 | | 5 | (8.8) | 3 | | 2 | | (3.5) | 2 | | 1 | (1.8) | | |
| Endocrine disorders | 10 | | 9 | (15.8) | 8 | | 7 | | (12.3) | 0 | | 0 | (0.0) | | |
| Eye disorders | 6 | | 6 | (10.5) | 4 | | 4 | | (7.0) | 2 | | 2 | (3.5) | | |
| Gastrointestinal disorders | 12 | | 10 | (17.5) | 9 | | 9 | | (15.8) | 2 | | 2 | (3.5) | | |
| General symptoms: pain, fatigue, edema | 30 | | 26 | (45.6) | 13 | | 13 | | (22.8) | 0 | | 0 | (0.0) | | |
| Infections and infestations | 10 | | 9 | (15.8) | 9 | | 8 | | (14.0) | 8 | | 6 | (10.5) | | |
| Injury, poisoning and procedural complications | 14 | | 12 | (21.1) | 13 | | 12 | | (21.1) | 5 | | 5 | (8.8) | | |
| Laboratory, imaging and functional findings | 15 | | 15 | (26.3) | 1 | | 1 | | (1.8) | 0 | | 0 | (0.0) | | |
| Metabolism and nutrition disorders | 33 | | 28 | (49.1) | 27 | | 25 | | (43.9) | 8 | | 8 | (14.0) | | |
| Musculoskeletal and connective tissue disorders | 92 | | 44 | (77.2) | 33 | | 35 | | (61.4) | 4 | | 4 | (7.0) | | |
| Neoplasms benign, malignant and unspecified (incl cysts and polyps) | 12 | | 9 | (15.8) | 9 | | 8 | | (14.0) | 7 | | 7 | (12.3) | | |
| Nervous system disorders | 23 | | 17 | (29.8) | 10 | | 7 | | (12.3) | 5 | | 4 | (7.0) | | |
| Pregnancy, puerperium and perinatal conditions | 3 | | 2 | (3.5) | 3 | | 2 | | (3.5) | 3 | | 2 | (3.5) | | |
| Psychiatric disorders | 47 | | 28 | (49.1) | 17 | | 13 | | (22.8) | 5 | | 5 | (8.8) | | |
| Renal and urinary disorders | 1 | | 1 | (1.8) | 0 | | 0 | | (0.0) | 0 | | 0 | (0.0) | | |
| Reproductive system and breast disorders | 5 | | 5 | (8.8) | 4 | | 4 | | (7.0) | 0 | | 0 | (0.0) | | |
| Respiratory, thoracic and mediastinal disorders | 14 | | 10 | (17.5) | 8 | | 7 | | (12.3) | 6 | | 6 | (10.5) | | |
| Skin and subcutaneous tissue disorders | 5 | | 4 | (7.0) | 1 | | 1 | | (1.8) | 0 | | 0 | (0.0) | | |
| Surgical and medical procedures | 17 | | 15 | (26.3) | 13 | | 12 | | (21.1) | 6 | | 5 | (8.8) | | |
| Vascular disorders | 18 | | 16 | (28.1) | 15 | | 14 | | (24.6) | 5 | | 5 | (8.8) | | |
| *All SOCs combined* | *389* | | *56* | *(98.2)* | *202* | | *54* | | *(94.7)* | *68* | | *21* | *(36.8)* | | |
|  | **Lymphoma (N=35)** | | | | | | | | | | | | | | |
|  | Events any grade (1-4) | | | | Events grades 2+ (2-4) | | | | | Events grades 3+ (3-4) | | | | | |
|  | N events^a^ | | N persons^b^ | (%)^c^ | N events^a^ | | N persons^b^ | | (%)^c^ | N events^a^ | | N persons^b^ | (%)^c^ | | |
| Blood and lymphatic system disorders | 1 | | 1 | (2.9) | 0 | | 0 | | (0.0) | 0 | | 0 | (0.0) | | |
| Cardiac disorders | 7 | | 6 | (17.1) | 0 | | 0 | | (0.0) | 0 | | 0 | (0.0) | | |
| Congenital, familial and genetic disorders | 1 | | 1 | (2.9) | 0 | | 0 | | (0.0) | 0 | | 0 | (0.0) | | |
| Ear and labyrinth disorders | 0 | | 0 | (0.0) | 0 | | 0 | | (0.0) | 0 | | 0 | (0.0) | | |
| Endocrine disorders | 7 | | 6 | (17.1) | 4 | | 3 | | (8.6) | 0 | | 0 | (0.0) | | |
| Eye disorders | 3 | | 3 | (8.6) | 3 | | 3 | | (8.6) | 2 | | 2 | (5.7) | | |
| Gastrointestinal disorders | 7 | | 6 | (17.1) | 5 | | 4 | | (11.4) | 3 | | 2 | (5.7) | | |
| General symptoms: pain, fatigue, edema | 11 | | 9 | (25.7) | 6 | | 5 | | (14.3) | 1 | | 1 | (2.9) | | |
| Infections and infestations | 2 | | 2 | (5.7) | 2 | | 2 | | (5.7) | 2 | | 2 | (5.7) | | |
| Injury, poisoning and procedural complications | 12 | | 10 | (28.6) | 10 | | 9 | | (25.7) | 7 | | 6 | (17.1) | | |
| Laboratory, imaging and functional findings | 11 | | 9 | (25.7) | 4 | | 4 | | (11.4) | 2 | | 2 | (5.7) | | |
| Metabolism and nutrition disorders | 16 | | 14 | (40.0) | 11 | | 10 | | (28.6) | 3 | | 3 | (8.6) | | |
| Musculoskeletal and connective tissue disorders | 40 | | 26 | (74.3) | 12 | | 8 | | (22.9) | 3 | | 3 | (8.6) | | |
| Neoplasms benign, malignant and unspecified (incl cysts and polyps) | 8 | | 5 | (14.3) | 4 | | 4 | | (11.4) | 2 | | 2 | (5.7) | | |
| Nervous system disorders | 14 | | 12 | (34.3) | 4 | | 4 | | (11.4) | 0 | | 0 | (0.0) | | |
| Pregnancy, puerperium and perinatal conditions | 1 | | 1 | (2.9) | 1 | | 1 | | (2.9) | 1 | | 1 | (2.9) | | |
| Psychiatric disorders | 19 | | 16 | (45.7) | 3 | | 2 | | (5.7) | 0 | | 0 | (0.0) | | |
| Renal and urinary disorders | 2 | | 2 | (5.7) | 2 | | 2 | | (5.7) | 2 | | 2 | (5.7) | | |
| Reproductive system and breast disorders | 7 | | 7 | (20.0) | 7 | | 7 | | (20.0) | 0 | | 0 | (0.0) | | |
| Respiratory, thoracic and mediastinal disorders | 8 | | 7 | (20.0) | 5 | | 5 | | (14.3) | 2 | | 2 | (5.7) | | |
| Skin and subcutaneous tissue disorders | 3 | | 3 | (8.6) | 2 | | 2 | | (5.7) | 0 | | 0 | (0.0) | | |
| Surgical and medical procedures | 12 | | 9 | (25.7) | 11 | | 8 | | (22.9) | 6 | | 5 | (14.3) | | |
| Vascular disorders | 11 | | 10 | (28.6) | 11 | | 10 | | (28.6) | 0 | | 0 | (0.0) | | |
| *All SOCs combined* | *203* | | *35* | *(100.0)* | *107* | | *33* | | *(94.3)* | *36* | | *12* | *(34.3)* | | |
|  | **CNS (N=18)** | | | | | | | | | | | | | | |
|  | Events any grade (1-4) | | | | Events grades 2+ (2-4) | | | | | Events grades 3+ (3-4) | | | | | |
|  | N events^a^ | | N persons^b^ | (%)^c^ | N events^a^ | | N persons^b^ | | (%)^c^ | N events^a^ | | N persons^b^ | (%)^c^ | | |
| Blood and lymphatic system disorders | 0 | | 0 | (0.0) | 0 | | 0 | | (0.0) | 0 | | 0 | (0.0) | | |
| Cardiac disorders | 2 | | 2 | (11.1) | 0 | | 0 | | (0.0) | 0 | | 0 | (0.0) | | |
| Congenital, familial and genetic disorders | 0 | | 0 | (0.0) | 0 | | 0 | | (0.0) | 0 | | 0 | (0.0) | | |
| Ear and labyrinth disorders | 6 | | 6 | (33.3) | 1 | | 1 | | (5.6) | 1 | | 1 | (5.6) | | |
| Endocrine disorders | 12 | | 8 | (44.4) | 9 | | 8 | | (44.4) | 2 | | 2 | (11.1) | | |
| Eye disorders | 5 | | 4 | (22.2) | 5 | | 4 | | (22.2) | 1 | | 1 | (5.6) | | |
| Gastrointestinal disorders | 2 | | 2 | (11.1) | 0 | | 0 | | (0.0) | 0 | | 0 | (0.0) | | |
| General symptoms: pain, fatigue, edema | 6 | | 6 | (33.3) | 3 | | 3 | | (16.7) | 0 | | 0 | (0.0) | | |
| Infections and infestations | 2 | | 2 | (11.1) | 1 | | 1 | | (5.6) | 1 | | 1 | (5.6) | | |
| Injury, poisoning and procedural complications | 4 | | 4 | (22.2) | 2 | | 2 | | (11.1) | 2 | | 2 | (11.1) | | |
| Laboratory, imaging and functional findings | 7 | | 6 | (33.3) | 6 | | 5 | | (27.8) | 0 | | 0 | (0.0) | | |
| Metabolism and nutrition disorders | 10 | | 9 | (50.0) | 8 | | 8 | | (44.4) | 5 | | 5 | (27.8) | | |
| Musculoskeletal and connective tissue disorders | 29 | | 14 | (77.8) | 16 | | 10 | | (55.6) | 1 | | 1 | (5.6) | | |
| Neoplasms benign, malignant and unspecified (incl cysts and polyps) | 0 | | 0 | (0.0) | 0 | | 0 | | (0.0) | 0 | | 0 | (0.0) | | |
| Nervous system disorders | 26 | | 16 | (88.9) | 8 | | 6 | | (33.3) | 2 | | 2 | (11.1) | | |
| Pregnancy, puerperium and perinatal conditions | 0 | | 0 | (0.0) | 0 | | 0 | | (0.0) | 0 | | 0 | (0.0) | | |
| Psychiatric disorders | 17 | | 8 | (44.4) | 6 | | 4 | | (22.2) | 1 | | 1 | (5.6) | | |
| Renal and urinary disorders | 0 | | 0 | (0.0) | 0 | | 0 | | (0.0) | 0 | | 0 | (0.0) | | |
| Reproductive system and breast disorders | 0 | | 0 | (0.0) | 0 | | 0 | | (0.0) | 0 | | 0 | (0.0) | | |
| Respiratory, thoracic and mediastinal disorders | 6 | | 6 | (33.3) | 2 | | 2 | | (11.1) | 0 | | 0 | (0.0) | | |
| Skin and subcutaneous tissue disorders | 1 | | 1 | (5.6) | 0 | | 0 | | (0.0) | 0 | | 0 | (0.0) | | |
| Surgical and medical procedures | 9 | | 8 | (44.4) | 8 | | 7 | | (38.9) | 4 | | 4 | (22.2) | | |
| Vascular disorders | 2 | | 2 | (11.1) | 2 | | 2 | | (11.1) | 0 | | 0 | (0.0) | | |
| *All SOCs combined* | *147* | | *18* | *(100.0)* | *77* | | *17* | | *(94.4)* | *20* | | *5* | *(27.8)* | | |
|  | **Bone / Soft Tissue Sarcoma (N=19)** | | | | | | | | | | | | | | |
|  | Events any grade (1-4) | | | | Events grades 2+ (2-4) | | | | | Events grades 3+ (3-4) | | | | | |
|  | N events^a^ | | N persons^b^ | (%)^c^ | N events^a^ | | N persons^b^ | | (%)^c^ | N events^a^ | | N persons^b^ | (%)^c^ | | |
| Blood and lymphatic system disorders | 0 | | 0 | (0.0) | 0 | | 0 | | (0.0) | 0 | | 0 | (0.0) | | |
| Cardiac disorders | 5 | | 4 | (21.1) | 3 | | 3 | | (15.8) | 1 | | 1 | (5.3) | | |
| Congenital, familial and genetic disorders | 1 | | 1 | (5.3) | 1 | | 1 | | (5.3) | 1 | | 1 | (5.3) | | |
| Ear and labyrinth disorders | 1 | | 1 | (5.3) | 0 | | 0 | | (0.0) | 0 | | 0 | (0.0) | | |
| Endocrine disorders | 7 | | 7 | (36.8) | 5 | | 5 | | (26.3) | 0 | | 0 | (0.0) | | |
| Eye disorders | 3 | | 2 | (10.5) | 3 | | 2 | | (10.5) | 1 | | 1 | (5.3) | | |
| Gastrointestinal disorders | 5 | | 4 | (21.1) | 2 | | 2 | | (10.5) | 1 | | 1 | (5.3) | | |
| General symptoms: pain, fatigue, edema | 8 | | 6 | (31.6) | 3 | | 3 | | (15.8) | 1 | | 1 | (5.3) | | |
| Infections and infestations | 4 | | 4 | (21.1) | 4 | | 4 | | (21.1) | 4 | | 4 | (21.1) | | |
| Injury, poisoning and procedural complications | 13 | | 11 | (57.9) | 13 | | 11 | | (57.9) | 7 | | 7 | (36.8) | | |
| Laboratory, imaging and functional findings | 4 | | 4 | (21.1) | 1 | | 1 | | (5.3) | 0 | | 0 | (0.0) | | |
| Metabolism and nutrition disorders | 3 | | 3 | (15.8) | 3 | | 3 | | (15.8) | 0 | | 0 | (0.0) | | |
| Musculoskeletal and connective tissue disorders | 42 | | 17 | (89.5) | 24 | | 13 | | (68.4) | 5 | | 5 | (26.3) | | |
| Neoplasms benign, malignant and unspecified (incl cysts and polyps) | 5 | | 4 | (21.1) | 3 | | 3 | | (15.8) | 2 | | 2 | (10.5) | | |
| Nervous system disorders | 15 | | 8 | (42.1) | 8 | | 6 | | (31.6) | 2 | | 2 | (10.5) | | |
| Pregnancy, puerperium and perinatal conditions | 0 | | 0 | (0.0) | 0 | | 0 | | (0.0) | 0 | | 0 | (0.0) | | |
| Psychiatric disorders | 12 | | 10 | (52.6) | 3 | | 3 | | (15.8) | 0 | | 0 | (0.0) | | |
| Renal and urinary disorders | 0 | | 0 | (0.0) | 0 | | 0 | | (0.0) | 0 | | 0 | (0.0) | | |
| Reproductive system and breast disorders | 6 | | 4 | (21.1) | 4 | | 3 | | (15.8) | 3 | | 3 | (15.8) | | |
| Respiratory, thoracic and mediastinal disorders | 6 | | 4 | (21.1) | 2 | | 2 | | (10.5) | 1 | | 1 | (5.3) | | |
| Skin and subcutaneous tissue disorders | 1 | | 1 | (5.3) | 1 | | 1 | | (5.3) | 0 | | 0 | (0.0) | | |
| Surgical and medical procedures | 14 | | 10 | (52.6) | 14 | | 10 | | (52.6) | 8 | | 5 | (26.3) | | |
| Vascular disorders | 2 | | 2 | (10.5) | 1 | | 1 | | (5.3) | 1 | | 1 | (5.3) | | |
| *All SOCs combined* | *155* | | *19* | *(100.0)* | *97* | | *18* | | *(94.7)* | *37* | | *11* | *(57.9)* | | |
|  | **Other (N=34)** | | | | | | | | | | | | | | |
|  | Events any grade (1-4) | | | | Events grades 2+ (2-4) | | | | | Events grades 3+ (3-4) | | | | | |
|  | N events^a^ | | N persons^b^ | (%)^c^ | N events^a^ | | N persons^b^ | | (%)^c^ | N events^a^ | | N persons^b^ | (%)^c^ | | |
| Blood and lymphatic system disorders | 0 | | 0 | (0.0) | 0 | | 0 | | (0.0) | 0 | | 0 | (0.0) | | |
| Cardiac disorders | 15 | | 13 | (38.2) | 6 | | 6 | | (17.6) | 2 | | 2 | (5.9) | | |
| Congenital, familial and genetic disorders | 1 | | 1 | (2.9) | 1 | | 1 | | (2.9) | 1 | | 1 | (2.9) | | |
| Ear and labyrinth disorders | 6 | | 5 | (14.7) | 3 | | 2 | | (5.9) | 1 | | 1 | (2.9) | | |
| Endocrine disorders | 23 | | 11 | (32.4) | 21 | | 10 | | (29.4) | 5 | | 4 | (11.8) | | |
| Eye disorders | 4 | | 4 | (11.8) | 4 | | 4 | | (11.8) | 2 | | 2 | (5.9) | | |
| Gastrointestinal disorders | 4 | | 4 | (11.8) | 4 | | 4 | | (11.8) | 3 | | 3 | (8.8) | | |
| General symptoms: pain, fatigue, edema | 14 | | 13 | (38.2) | 3 | | 3 | | (8.8) | 0 | | 0 | (0.0) | | |
| Infections and infestations | 5 | | 5 | (14.7) | 5 | | 5 | | (14.7) | 4 | | 4 | (11.8) | | |
| Injury, poisoning and procedural complications | 20 | | 14 | (41.2) | 19 | | 13 | | (38.2) | 8 | | 5 | (14.7) | | |
| Laboratory, imaging and functional findings | 8 | | 6 | (17.6) | 4 | | 4 | | (11.8) | 0 | | 0 | (0.0) | | |
| Metabolism and nutrition disorders | 13 | | 11 | (32.4) | 7 | | 6 | | (17.6) | 3 | | 2 | (5.9) | | |
| Musculoskeletal and connective tissue disorders | 57 | | 29 | (85.3) | 15 | | 12 | | (35.3) | 1 | | 1 | (2.9) | | |
| Neoplasms benign, malignant and unspecified (incl cysts and polyps) | 13 | | 8 | (23.5) | 10 | | 5 | | (14.7) | 8 | | 3 | (8.8) | | |
| Nervous system disorders | 22 | | 13 | (38.2) | 11 | | 7 | | (20.6) | 7 | | 6 | (17.6) | | |
| Pregnancy, puerperium and perinatal conditions | 1 | | 1 | (2.9) | 1 | | 1 | | (2.9) | 1 | | 1 | (2.9) | | |
| Psychiatric disorders | 27 | | 18 | (52.9) | 6 | | 6 | | (17.6) | 0 | | 0 | (0.0) | | |
| Renal and urinary disorders | 4 | | 3 | (8.8) | 4 | | 3 | | (8.8) | 3 | | 2 | (5.9) | | |
| Reproductive system and breast disorders | 4 | | 4 | (11.8) | 2 | | 2 | | (5.9) | 0 | | 0 | (0.0) | | |
| Respiratory, thoracic and mediastinal disorders | 11 | | 9 | (26.5) | 6 | | 5 | | (14.7) | 1 | | 1 | (2.9) | | |
| Skin and subcutaneous tissue disorders | 3 | | 3 | (8.8) | 2 | | 2 | | (5.9) | 0 | | 0 | (0.0) | | |
| Surgical and medical procedures | 14 | | 11 | (32.4) | 13 | | 10 | | (29.4) | 8 | | 5 | (14.7) | | |
| Vascular disorders | 7 | | 6 | (17.6) | 7 | | 6 | | (17.6) | 2 | | 2 | (5.9) | | |
| *All SOCs combined* | *276* | | *34* | *(100.0)* | *153* | | *31* | | *(91.2)* | *59* | | *14* | *(41.2)* | | |

Footnotes:
^a^ Total number of events reported in each SOC; a person can have several events.
^b^ Number of persons reporting at least one event within the respective SOC.
^c^ Percentage of persons who reported to have at least one event within the respective SOC.

Abbreviations: CTCAE, Common Terminology Criteria for Adverse Events; N, number; SOC, system organ class.

**Supplemental Table 2. Number of reported adverse events for any grade (1-4) and grades 2+ (2-4) and 3+ (3-4), by System Organ Class (SOC) and Preferred Term (PT) according to the Common Terminology Criteria for Adverse Events (CTCAE), *N*=163**

| **System Organ Class (SOC) and Preferred Term (PT)** | Events any grade (1-4) | | | Events grades 2+ (2-4) | | | | Events grades 3+ (3-4) | | | | |
| --- | --- | --- | --- | --- | --- | --- | --- | --- | --- | --- | --- | --- |
|  | N events^a^ | N persons^b^ | (%)^c^ | N events^a^ | N persons^b^ | (%)^c^ | | N events^a^ | N persons^b^ | | (%)^c^ | |
| **Blood and lymphatic system disorders** | 2 | 2 | 1.2 | 0 | 0 | | 0 | 0 | | 0 | | 0 |
| Anemia | 1 | 1 | 0.6 | 0 | 0 | | 0 | 0 | | 0 | | 0 |
| Other | 1 | 1 | 0.6 | 0 | 0 | | 0 | 0 | | 0 | | 0 |
| **Cardiac disorders** | 43 | 35 | 21.5 | 11 | 11 | | 6.7 | 3 | | 3 | | 1.8 |
| Aortic valve dissease | 1 | 1 | 0.6 | 0 | 0 | | 0.0 | 0 | | 0 | | 0.0 |
| Chest pain - cardiac | 9 | 9 | 5.5 | 2 | 2 | | 1.2 | 0 | | 0 | | 0.0 |
| Conduction disorder | 2 | 2 | 1.2 | 1 | 1 | | 0.6 | 1 | | 1 | | 0.6 |
| Cyanosis | 1 | 1 | 0.6 | 1 | 1 | | 0.6 | 0 | | 0 | | 0.0 |
| Other | 16 | 16 | 9.8 | 3 | 3 | | 1.8 | 0 | | 0 | | 0.0 |
| Palpitations | 10 | 10 | 6.1 | 0 | 0 | | 0.0 | 0 | | 0 | | 0.0 |
| Pericarditis | 2 | 2 | 1.2 | 2 | 2 | | 1.2 | 1 | | 1 | | 0.6 |
| Pulmonary valve disease | 1 | 1 | 0.6 | 1 | 1 | | 0.6 | 0 | | 0 | | 0.0 |
| Restrictive cardiomyopathy | 1 | 1 | 0.6 | 1 | 1 | | 0.6 | 1 | | 1 | | 0.6 |
| **Congenital, familial and genetic disorders** | 4 | 4 | 2.5 | 3 | 3 | | 1.8 | 2 | | 2 | | 1.2 |
| Other | 4 | 4 | 2.5 | 3 | 3 | | 1.8 | 2 | | 2 | | 1.2 |
| **Ear and labyrinth disorders** | 19 | 17 | 10.4 | 7 | 6 | | 3.7 | 4 | | 3 | | 1.8 |
| External ear inflammation | 1 | 1 | 0.6 | 0 | 0 | | 0.0 | 0 | | 0 | | 0.0 |
| Hearing impaired | 14 | 14 | 8.6 | 5 | 5 | | 3.1 | 3 | | 3 | | 1.8 |
| Other | 3 | 3 | 1.8 | 2 | 2 | | 1.2 | 1 | | 1 | | 0.6 |
| Otitis externa | 1 | 1 | 0.6 | 0 | 0 | | 0.0 | 0 | | 0 | | 0.0 |
| **Endocrine disorders** | 59 | 40 | 24.5 | 46 | 32 | | 19.6 | 6 | | 5 | | 3.1 |
| Adrenal insufficiency | 1 | 1 | 0.6 | 1 | 1 | | 0.6 | 0 | | 0 | | 0.0 |
| Delayed puberty | 8 | 8 | 4.9 | 8 | 8 | | 4.9 | 2 | | 2 | | 1.2 |
| Hyperglycemia | 2 | 2 | 1.2 | 2 | 2 | | 1.2 | 2 | | 2 | | 1.2 |
| Hypothyroidism | 19 | 19 | 11.7 | 17 | 17 | | 10.4 | 1 | | 1 | | 0.6 |
| Obesity | 3 | 3 | 1.8 | 3 | 3 | | 1.8 | 0 | | 0 | | 0.0 |
| Other | 25 | 23 | 14.1 | 15 | 15 | | 9.2 | 1 | | 1 | | 0.6 |
| Polymenorrhea | 1 | 1 | 0.6 | 0 | 0 | | 0.0 | 0 | | 0 | | 0.0 |
| **Eye disorders** | 21 | 19 | 11.7 | 19 | 17 | | 10.4 | 8 | | 7 | | 4.3 |
| Cataract | 4 | 4 | 2.5 | 3 | 3 | | 1.8 | 1 | | 1 | | 0.6 |
| Nystagmus | 3 | 3 | 1.8 | 3 | 3 | | 1.8 | 0 | | 0 | | 0.0 |
| Other | 6 | 6 | 3.7 | 6 | 6 | | 3.7 | 5 | | 5 | | 3.1 |
| Vision decreased | 8 | 8 | 4.9 | 7 | 7 | | 4.3 | 2 | | 2 | | 1.2 |
| **Gastrointestinal disorders** | 30 | 26 | 16.0 | 20 | 19 | | 11.7 | 9 | | 8 | | 4.908 |
| Cholecystitis | 1 | 1 | 0.6 | 1 | 1 | | 0.6 | 1 | | 1 | | 0.6 |
| Colonic obstruction | 1 | 1 | 0.6 | 1 | 1 | | 0.6 | 1 | | 1 | | 0.6 |
| Dental caries | 5 | 5 | 3.1 | 0 | 0 | | 0.0 | 0 | | 0 | | 0.0 |
| Diarrhea | 1 | 1 | 0.6 | 0 | 0 | | 0.0 | 0 | | 0 | | 0.0 |
| Dyspepsia | 5 | 5 | 3.1 | 4 | 4 | | 2.5 | 0 | | 0 | | 0.0 |
| Esophageal obstruction | 1 | 1 | 0.6 | 1 | 1 | | 0.6 | 1 | | 1 | | 0.6 |
| Esophagitis | 1 | 1 | 0.6 | 1 | 1 | | 0.6 | 0 | | 0 | | 0.0 |
| Gastric ulcer | 1 | 1 | 0.6 | 1 | 1 | | 0.6 | 1 | | 1 | | 0.6 |
| Gastritis | 2 | 2 | 1.2 | 2 | 2 | | 1.2 | 0 | | 0 | | 0.0 |
| Ileal obstruction | 2 | 2 | 1.2 | 2 | 2 | | 1.2 | 2 | | 2 | | 1.2 |
| Lower gastrointestinal hemorrhage | 1 | 1 | 0.6 | 1 | 1 | | 0.6 | 0 | | 0 | | 0.0 |
| Other | 7 | 7 | 4.3 | 5 | 5 | | 3.1 | 3 | | 3 | | 1.8 |
| Periodontal disease | 1 | 1 | 0.6 | 0 | 0 | | 0.0 | 0 | | 0 | | 0.0 |
| Renal calculi | 1 | 1 | 0.6 | 1 | 1 | | 0.6 | 0 | | 0 | | 0.0 |
| **General symptoms: pain, fatigue, edema** | 69 | 60 | 36.8 | 28 | 27 | | 16.6 | 2 | | 2 | | 1.2 |
| Edema limbs | 13 | 13 | 8.0 | 0 | 0 | | 0.0 | 0 | | 0 | | 0.0 |
| Fatigue | 52 | 52 | 31.9 | 25 | 25 | | 15.3 | 0 | | 0 | | 0.0 |
| Localized edema | 1 | 1 | 0.6 | 1 | 1 | | 0.6 | 0 | | 0 | | 0.0 |
| Non cardiac chest pain | 1 | 1 | 0.6 | 0 | 0 | | 0.0 | 0 | | 0 | | 0.0 |
| Pain | 2 | 2 | 1.2 | 2 | 2 | | 1.2 | 2 | | 2 | | 1.2 |
| **Infections and infestations** | 22 | 20 | 12.3 | 20 | 17 | | 10.4 | 19 | | 17 | | 10.4 |
| Bronchial infection | 2 | 2 | 1.2 | 2 | 2 | | 1.2 | 2 | | 2 | | 1.2 |
| Epstein-Barr virus infection reactivation | 1 | 1 | 0.6 | 1 | 1 | | 0.6 | 0 | | 0 | | 0.0 |
| Joint infection | 2 | 2 | 1.2 | 2 | 2 | | 1.2 | 2 | | 2 | | 1.2 |
| Lung infection | 2 | 2 | 1.2 | 2 | 2 | | 1.2 | 2 | | 2 | | 1.2 |
| Lymph gland infection | 1 | 1 | 0.6 | 1 | 1 | | 0.6 | 1 | | 1 | | 0.6 |
| Meningitis | 3 | 3 | 1.8 | 3 | 3 | | 1.8 | 3 | | 3 | | 1.8 |
| Other | 2 | 2 | 1.2 | 1 | 2 | | 1.2 | 1 | | 1 | | 0.6 |
| Penile infection | 1 | 1 | 0.6 | 0 | 0 | | 0.0 | 0 | | 0 | | 0.0 |
| Pleural infection | 1 | 1 | 0.6 | 1 | 1 | | 0.6 | 1 | | 1 | | 0.6 |
| Salivary gland infection | 1 | 1 | 0.6 | 1 | 1 | | 0.6 | 1 | | 1 | | 0.6 |
| Scrotal infection | 1 | 1 | 0.6 | 1 | 1 | | 0.6 | 1 | | 1 | | 0.6 |
| Sinusitis | 1 | 1 | 0.6 | 1 | 1 | | 0.6 | 1 | | 1 | | 0.6 |
| Skin infection | 1 | 1 | 0.6 | 1 | 1 | | 0.6 | 1 | | 1 | | 0.6 |
| Soft tissue infection | 1 | 1 | 0.6 | 1 | 1 | | 0.6 | 1 | | 1 | | 0.6 |
| Urinary tract infection | 1 | 1 | 0.6 | 1 | 1 | | 0.6 | 1 | | 1 | | 0.6 |
| Wound complication | 1 | 1 | 0.6 | 1 | 1 | | 0.6 | 1 | | 1 | | 0.6 |
| **Injury, poisoning and procedural complications** | 63 | 51 | 31.3 | 57 | 47 | | 28.8 | 29 | | 25 | | 15.3 |
| Ankle fracture | 1 | 1 | 0.6 | 1 | 1 | | 0.6 | 0 | | 0 | | 0.0 |
| Dermatitis radiation | 1 | 1 | 0.6 | 1 | 1 | | 0.6 | 0 | | 0 | | 0.0 |
| Fracture | 33 | 33 | 20.2 | 31 | 26 | | 16.0 | 15 | | 11 | | 6.7 |
| Intraoperative neurological injury | 1 | 1 | 0.6 | 0 | 0 | | 0.0 | 0 | | 0 | | 0.0 |
| Other | 17 | 17 | 10.4 | 14 | 14 | | 8.6 | 7 | | 7 | | 4.3 |
| Spinal fracture | 1 | 1 | 0.6 | 1 | 1 | | 0.6 | 0 | | 0 | | 0.0 |
| Vascular access complication | 1 | 1 | 0.6 | 1 | 1 | | 0.6 | 1 | | 1 | | 0.6 |
| Wound complication | 1 | 1 | 0.6 | 1 | 1 | | 0.6 | 1 | | 1 | | 0.6 |
| Wrist fracture | 7 | 7 | 4.3 | 7 | 7 | | 4.3 | 5 | | 5 | | 3.1 |
| **Laboratory, imaging and functional findings** | 46 | 42 | 25.8 | 16 | 15 | | 9.2 | 2 | | 2 | | 1.2 |
| Cholesterol high | 7 | 7 | 4.3 | 2 | 2 | | 1.2 | 0 | | 0 | | 0.0 |
| Electrocardiogram QT corrected interval prolonged | 2 | 2 | 1.2 | 0 | 0 | | 0.0 | 0 | | 0 | | 0.0 |
| Forced expiratory volume decreased | 26 | 26 | 16.0 | 5 | 5 | | 3.1 | 2 | | 2 | | 1.2 |
| Growth hormone abnormal | 7 | 7 | 4.3 | 7 | 7 | | 4.3 | 0 | | 0 | | 0.0 |
| Other | 3 | 3 | 1.8 | 2 | 2 | | 1.2 | 0 | | 0 | | 0.0 |
| Weight loss | 1 | 1 | 0.6 | 0 | 0 | | 0.0 | 0 | | 0 | | 0.0 |
| **Metabolism and nutrition disorders** | 74 | 65 | 39.9 | 55 | 52 | | 31.9 | 19 | | 18 | | 11.0 |
| Hyperglycemia | 15 | 15 | 9.2 | 5 | 5 | | 3.1 | 4 | | 4 | | 2.5 |
| Iron overload | 1 | 1 | 0.6 | 1 | 1 | | 0.6 | 1 | | 1 | | 0.6 |
| Obesity | 51 | 51 | 31.3 | 49 | 49 | | 30.1 | 14 | | 14 | | 8.6 |
| Other | 7 | 7 | 4.3 | 0 | 0 | | 0.0 | 0 | | 0 | | 0.0 |
| **Musculoskeletal and connective tissue disorders** | 260 | 130 | 79.8 | 100 | 69 | | 42.3 | 14 | | 14 | | 8.6 |
| Arthralgia | 10 | 10 | 6.1 | 3 | 3 | | 1.8 | 0 | | 0 | | 0.0 |
| Arthritis | 6 | 6 | 3.7 | 2 | 2 | | 1.2 | 1 | | 1 | | 0.6 |
| Avascular necrosis | 1 | 1 | 0.6 | 1 | 1 | | 0.6 | 0 | | 0 | | 0.0 |
| Back pain | 27 | 27 | 16.6 | 9 | 9 | | 5.5 | 2 | | 2 | | 1.2 |
| Brachial plexopathy | 1 | 1 | 0.6 | 1 | 1 | | 0.6 | 1 | | 1 | | 0.6 |
| Chest wall pain | 2 | 2 | 1.2 | 0 | 0 | | 0.0 | 0 | | 0 | | 0.0 |
| Exostosis | 2 | 2 | 1.2 | 1 | 1 | | 0.6 | 0 | | 0 | | 0.0 |
| Generalized muscle weakness | 24 | 24 | 14.7 | 24 | 24 | | 14.7 | 0 | | 0 | | 0.0 |
| Joint range of motion decreased | 14 | 14 | 8.6 | 3 | 3 | | 1.8 | 2 | | 2 | | 1.2 |
| Kyphosis | 1 | 1 | 0.6 | 1 | 1 | | 0.6 | 1 | | 1 | | 0.6 |
| Muscle weakness left-sided | 1 | 1 | 0.6 | 1 | 1 | | 0.6 | 0 | | 0 | | 0.0 |
| Muscle weakness right-sided | 1 | 1 | 0.6 | 1 | 1 | | 0.6 | 0 | | 0 | | 0.0 |
| Muscle weakness trunk | 1 | 1 | 0.6 | 1 | 1 | | 0.6 | 0 | | 0 | | 0.0 |
| Muscle weakness upper limb | 4 | 4 | 2.5 | 3 | 3 | | 1.8 | 1 | | 1 | | 0.6 |
| Musculoskeletal deformity | 2 | 2 | 1.2 | 1 | 1 | | 0.6 | 1 | | 1 | | 0.6 |
| Neck pain | 3 | 3 | 1.8 | 1 | 1 | | 0.6 | 0 | | 0 | | 0.0 |
| Osteoporosis | 101 | 101 | 62.0 | 13 | 13 | | 8.0 | 0 | | 0 | | 0.0 |
| Other | 22 | 22 | 13.5 | 20 | 19 | | 11.7 | 3 | | 3 | | 1.8 |
| Pain | 1 | 1 | 0.6 | 0 | 0 | | 0.0 | 0 | | 0 | | 0.0 |
| Pain in extremity | 20 | 20 | 12.3 | 4 | 4 | | 2.5 | 0 | | 0 | | 0.0 |
| Rotator cuff injury | 1 | 1 | 0.6 | 0 | 0 | | 0.0 | 0 | | 0 | | 0.0 |
| Scoliosis | 13 | 13 | 8.0 | 10 | 10 | | 6.1 | 3 | | 3 | | 1.8 |
| Unequal limb length | 2 | 2 | 1.2 | 0 | 0 | | 0.0 | 0 | | 0 | | 0.0 |
| **Neoplasms benign, malignant and unspecified (incl cysts and polyps)** | 38 | 26 | 16.0 | 26 | 20 | | 12.3 | 19 | | 14 | | 8.6 |
| Treatment related secondary malignancy | 1 | 1 | 0.6 | 1 | 1 | | 0.6 | 1 | | 1 | | 0.6 |
| Other | 37 | 30 | 18.4 | 25 | 19 | | 11.7 | 18 | | 13 | | 8.0 |
| **Nervous system disorders** | 100 | 66 | 40.5 | 41 | 32 | | 19.6 | 14 | | 14 | | 8.6 |
| Anosmia | 1 | 1 | 0.6 | 0 | 0 | | 0.0 | 0 | | 0 | | 0.0 |
| Ataxia | 22 | 21 | 12.9 | 5 | 4 | | 2.5 | 0 | | 0 | | 0.0 |
| Concentration impairment | 2 | 2 | 1.2 | 2 | 2 | | 1.2 | 0 | | 0 | | 0.0 |
| Facial nerve disorder | 2 | 2 | 1.2 | 2 | 2 | | 1.2 | 1 | | 1 | | 0.6 |
| Headache | 12 | 12 | 7.4 | 8 | 8 | | 4.9 | 1 | | 1 | | 0.6 |
| Hypersomnia | 1 | 1 | 0.6 | 1 | 1 | | 0.6 | 1 | | 1 | | 0.6 |
| Intracranial hemorrhage | 1 | 1 | 0.6 | 1 | 1 | | 0.6 | 1 | | 1 | | 0.6 |
| Movements involuntary | 1 | 1 | 0.6 | 0 | 0 | | 0.0 | 0 | | 0 | | 0.0 |
| Other | 7 | 7 | 4.3 | 6 | 6 | | 3.7 | 1 | | 1 | | 0.6 |
| Paresthesia | 2 | 2 | 1.2 | 1 | 1 | | 0.6 | 0 | | 0 | | 0.0 |
| Peripheral motor neuropathy | 4 | 4 | 2.5 | 2 | 2 | | 1.2 | 0 | | 0 | | 0.0 |
| Peripheral sensory neuropathy | 32 | 29 | 17.8 | 3 | 3 | | 1.8 | 0 | | 0 | | 0.0 |
| Seizure | 4 | 4 | 2.5 | 4 | 4 | | 2.5 | 3 | | 3 | | 1.8 |
| Stroke | 3 | 3 | 1.8 | 3 | 3 | | 1.8 | 3 | | 3 | | 1.8 |
| Syncope | 3 | 3 | 1.8 | 3 | 3 | | 1.8 | 3 | | 3 | | 1.8 |
| Trigeminal nerve disorder | 3 | 3 | 1.8 | 0 | 0 | | 0.0 | 0 | | 0 | | 0.0 |
| **Pregnancy, puerperium and perinatal conditions** | 5 | 4 | 2.5 | 5 | 4 | | 2.5 | 5 | | 4 | | 2.5 |
| Pregnancy loss | 4 | 4 | 2.5 | 4 | 4 | | 2.5 | 4 | | 4 | | 2.5 |
| Other | 1 | 1 | 0.6 | 1 | 1 | | 0.6 | 1 | | 1 | | 0.6 |
| **Psychiatric disorders** | 122 | 80 | 49.1 | 35 | 28 | | 17.2 | 6 | | 6 | | 3.7 |
| Anxiety | 15 | 15 | 9.2 | 3 | 3 | | 1.8 | 0 | | 0 | | 0.0 |
| Depression | 28 | 28 | 17.2 | 7 | 7 | | 4.3 | 0 | | 0 | | 0.0 |
| Insomnia | 67 | 67 | 41.1 | 18 | 18 | | 11.0 | 5 | | 5 | | 3.1 |
| Other | 11 | 11 | 6.7 | 6 | 6 | | 3.7 | 1 | | 1 | | 0.6 |
| Suicidal ideation | 1 | 1 | 0.6 | 1 | 1 | | 0.6 | 0 | | 0 | | 0.0 |
| **Renal and urinary disorders** | 7 | 6 | 3.7 | 6 | 5 | | 3.1 | 5 | | 4 | | 2.5 |
| Chronic kidney disease | 3 | 3 | 1.8 | 3 | 3 | | 1.8 | 3 | | 3 | | 1.8 |
| Renal calculi | 1 | 1 | 0.6 | 1 | 1 | | 0.6 | 1 | | 1 | | 0.6 |
| Urinary incontinence | 1 | 1 | 0.6 | 1 | 1 | | 0.6 | 0 | | 0 | | 0.0 |
| Urinary tract obstruction | 1 | 1 | 0.6 | 1 | 1 | | 0.6 | 1 | | 1 | | 0.6 |
| Urinary tract pain | 1 | 1 | 0.6 | 0 | 0 | | 0.0 | 0 | | 0 | | 0.0 |
| **Reproductive system and breast disorders** | 22 | 20 | 12.3 | 17 | 16 | | 9.8 | 3 | | 3 | | 1.8 |
| Azoospermia | 7 | 7 | 4.3 | 7 | 7 | | 4.3 | 0 | | 0 | | 0.0 |
| Dysmenorrhea | 2 | 2 | 1.2 | 1 | 1 | | 0.6 | 0 | | 0 | | 0.0 |
| Gynecomastia | 1 | 1 | 0.6 | 1 | 1 | | 0.6 | 1 | | 1 | | 0.6 |
| Irregular menstruation | 3 | 3 | 1.8 | 2 | 2 | | 1.2 | 2 | | 2 | | 1.2 |
| Menorrhagia | 2 | 2 | 1.2 | 1 | 1 | | 0.6 | 0 | | 0 | | 0.0 |
| Other | 4 | 4 | 2.5 | 2 | 2 | | 1.2 | 0 | | 0 | | 0.0 |
| Premature menopause | 1 | 1 | 0.6 | 1 | 1 | | 0.6 | 0 | | 0 | | 0.0 |
| Prostatic obstruction | 1 | 1 | 0.6 | 1 | 1 | | 0.6 | 0 | | 0 | | 0.0 |
| Testicular disorder | 1 | 1 | 0.6 | 1 | 1 | | 0.6 | 0 | | 0 | | 0.0 |
| **Respiratory, thoracic and mediastinal disorders** | 45 | 36 | 22.1 | 23 | 23 | | 14.1 | 10 | | 10 | | 6.1 |
| Bronchospasm | 1 | 1 | 0.6 | 1 | 1 | | 0.6 | 0 | | 0 | | 0.0 |
| Cough | 6 | 6 | 3.7 | 3 | 3 | | 1.8 | 0 | | 0 | | 0.0 |
| Dyspnea | 14 | 14 | 8.6 | 8 | 8 | | 4.9 | 7 | | 7 | | 4.3 |
| Other | 15 | 15 | 9.2 | 6 | 6 | | 3.7 | 1 | | 1 | | 0.6 |
| Pneumothorax | 2 | 2 | 1.2 | 2 | 2 | | 1.2 | 1 | | 1 | | 0.6 |
| Productive cough | 1 | 1 | 0.6 | 0 | 0 | | 0.0 | 0 | | 0 | | 0.0 |
| Sleep apnea | 2 | 2 | 1.2 | 1 | 1 | | 0.6 | 1 | | 1 | | 0.6 |
| Wheezing | 4 | 4 | 2.5 | 2 | 2 | | 1.2 | 0 | | 0 | | 0.0 |
| **Skin and subcutaneous tissue disorders** | 13 | 12 | 7.4 | 6 | 6 | | 3.7 | 0 | | 0 | | 0.0 |
| Eczema | 1 | 1 | 0.6 | 0 | 0 | | 0.0 | 0 | | 0 | | 0.0 |
| Other | 8 | 8 | 4.9 | 4 | 4 | | 2.5 | 0 | | 0 | | 0.0 |
| Pruritus | 1 | 1 | 0.6 | 1 | 1 | | 0.6 | 0 | | 0 | | 0.0 |
| Purpura | 1 | 1 | 0.6 | 0 | 0 | | 0.0 | 0 | | 0 | | 0.0 |
| Tooth discoloration | 1 | 1 | 0.6 | 0 | 0 | | 0.0 | 0 | | 0 | | 0.0 |
| Urticaria | 1 | 1 | 0.6 | 1 | 1 | | 0.6 | 0 | | 0 | | 0.0 |
| **Surgical and medical procedures** | 66 | 53 | 32.5 | 59 | 47 | | 28.8 | 32 | | 24 | | 14.7 |
| Other | 66 | 55 | 33.7 | 59 | 49 | | 30.1 | 32 | | 27 | | 16.6 |
| **Vascular disorders** | 40 | 36 | 22.1 | 36 | 33 | | 20.2 | 8 | | 8 | | 4.9 |
| Hypertension | 34 | 34 | 20.9 | 32 | 32 | | 19.6 | 5 | | 5 | | 3.1 |
| Other | 4 | 4 | 2.5 | 2 | 2 | | 1.2 | 1 | | 1 | | 0.6 |
| Thromboembolic event | 2 | 2 | 1.2 | 2 | 2 | | 1.2 | 2 | | 2 | | 1.2 |
| ***All SOCs combined*** | *1170* | *162* | *99.4* | *636* | *152* | | *93.252* | *220* | | *63* | | *38.65* |

^a^ Total number of events reported in each SOC; a person can have several events.
^b^ Number of persons reporting at least one event within the respective SOC.
^c^ Percentage of persons (from N=163) who reported to have at least one event within the respective SOC.

Abbreviations: CTCAE, Common Terminology Criteria for Adverse Events; N, number; PT, preferred term; SOC, system organ class.

**Supplemental Table 3, Association between physical fitness z-scores and prevalence of adverse events from Poisson regression models, *N=159-163^a^***

|  | **Basic Model 0^b^** | | | **Adjusted Model 1^c^** | | | **Adjusted Model 2^d^** | | |
| --- | --- | --- | --- | --- | --- | --- | --- | --- | --- |
|  | PRR | 95%CI | p-value | PRR | 95%CI | p-value | PRR | 95%CI | p-value |
| **Events of any grade** | | | | | | | | | |
| Aerobic capacity  [z-score for watt/kg bodyweight] | 0.80 | [0.76, 0.85] | <0.001 | 0.85 | [0.79, 0.91] | <0.001 | 0.84 | [0.79, 0.90] | <0.001 |
| Hand-grip strength [z-score for kg/kg bodyweight] | 0.93 | [0.89, 0.98] | 0.005 | 0.94 | [0.90, 1.00] | 0.021 | 0.92 | [0.88, 0.97] | 0.002 |
| Lower body endurance  [z-score for 1 repetition] | 0.87 | [0.83, 0.92] | <0.001 | 0.89 | [0.85, 0.94] | <0.001 | 0.89 | [0.85, 0.94] | <0.001 |
| Composite fitness score^e^ [per z-score] | 0.79 | [0.74, 0.85] | <0.001 | 0.83 | [0.77, 0.89] | <0.001 | 0.81 | [0.75, 0.88] | <0.001 |
| **Events grades 2+** | | | | | | | | | |
| Aerobic capacity | 0.80 | [0.74, 0.87] | <0.001 | 0.84 | [0.77, 0.92] | <0.001 | 0.85 | [0.77, 0.93] | 0.001 |
| Hand-grip strength | 0.98 | [0.92, 1.05] | 0.541 | 0.99 | [0.93, 1.06] | 0.725 | 0.96 | [0.90, 1.03] | 0.307 |
| Lower body endurance | 0.86 | [0.81, 0.92] | <0.001 | 0.88 | [0.82, 0.94] | <0.001 | 0.88 | [0.82, 0.95] | 0.001 |
| Composite fitness score | 0.81 | [0.74, 0.89] | <0.001 | 0.85 | [0.77, 0.94] | 0.002 | 0.84 | [0.76, 0.93] | 0.001 |
| **Events grades 3+** | | | | | | | | | |
| Aerobic capacity | 0.75 | [0.65, 0.85] | <0.001 | 0.81 | [0.69, 0.95] | 0.010 | 0.82 | [0.69, 0.96] | 0.015 |
| Hand-grip strength | 0.95 | [0.85, 1.06] | 0.368 | 0.98 | [0.88, 1.10] | 0.740 | 0.95 | [0.84, 1.07] | 0.367 |
| Lower body endurance | 0.78 | [0.70, 0.88] | <0.001 | 0.82 | [0.73, 0.92] | 0.001 | 0.83 | [0.74, 0.94] | 0.004 |
| Composite fitness score | 0.73 | [0.62, 0.85] | <0.001 | 0.89 | [0.68, 0.95] | 0.011 | 0.79 | [0.66, 0.94] | 0.009 |

Footnotes:
^a^ n=159 for aerobic capacity z-score, n=160 for hand-grip strength z-score, n=163 for lower body endurance z-score, n= 163 for composite fitness z-score
^b^ Basic Model 0: adjusted for age, sex, time since diagnosis.
^c^ Adjusted Model 1: Model 0 + BMI.
^d^ Adjusted Model 2: Model 1 + cancer types, anthracycline dose, steroid dose, location of radiotherapy. We calculated the cumulative anthracyclines dose as doxorubicin isotoxic equivalent dose (mg/m^2^) and the cumulative steroid dose as prednisone equivalent dose (mg/m^2^). Patients who did not receive steroid or anthracycline treatment, received 0 mg/m^2^ as their dose; 6 participants who received steroids but had missing information on cumulative dose were imputed with the median dose of all participants with steroid treatment.
^e^ Average z-score of aerobic capacity, hand-grip strength and lower body endurance.

Abbreviations: BMI, Body-Mass-Index; CI, Confidence Interval; PRR, Prevalence Rate Ratio.

**Supplemental** **Table 4, Association between aerobic capacity (maximal tests only) and of adverse events from Poisson regression models, *N=128-163^a^***

|  | **Basic Model 0^b^** | | | **Adjusted Model 1^c^** | | | **Adjusted Model 2^d^** | | |
| --- | --- | --- | --- | --- | --- | --- | --- | --- | --- |
|  | PRR | 95%CI | p-value | PRR | 95%CI | p-value | PRR | 95%CI | p-value |
| **Events of any grade** | | | | | | | | | |
| Aerobic capacity^e^  [per watt/kg bodyweight] | 0.67 | [0.59, 0.76] | <0.001 | 0.74 | [0.64, 0.85] | <0.001 | 0.76 | [0.65, 0.88] | <0.001 |
| Composite fitness z-score^f^ | 0.80 | [0.74, 0.87] | <0.001 | 0.85 | [0.78, 0.92] | <0.001 | 0.84 | [0.77, 0.92] | <0.001 |
| **Events grades 2+** | | | | | | | | | |
| Aerobic capacity | 0.69 | [0.59, 0.82] | <0.001 | 0.74 | [0.61, 0.89] | 0.002 | 0.80 | [0.65, 0.98] | 0.030 |
| Composite fitness z-score | 0.85 | [0.76, 0.95] | 0.004 | 0.88 | [0.78, 0.99] | 0.034 | 0.89 | [0.79, 1.01] | 0.077 |
| **Events grades 3+** | | | | | | | | | |
| Aerobic capacity | 0.62 | [0.46, 0.84] | 0.002 | 0.66 | [0.46, 0.93] | 0.018 | 0.73 | [0.51, 1.05] | 0.087 |
| Composite fitness z-score | 0.74 | [0.60, 0.90] | 0.003 | 0.78 | [0.63, 0.97] | 0.024 | 0.80 | [0.63, 1.00] | 0.051 |

Footnotes:
^a^ n=128 for aerobic capacity, n= 163 for composite fitness score ^b^ Basic Model 0: adjusted for age, sex, time since diagnosis.
^c^ Adjusted Model 1: Model 0 + BMI.
^d^ Adjusted Model 2: Model 1 + cancer types, anthracycline dose, steroid dose, location of radiotherapy. We calculated the cumulative anthracyclines dose as doxorubicin isotoxic equivalent dose (mg/m^2^) and the cumulative steroid dose as prednisone equivalent dose (mg/m^2^). Patients who did not receive steroid or anthracycline treatment, received 0 mg/m^2^ as their dose; 6 participants who received steroids but had missing information on cumulative dose were imputed with the median dose of all participants with steroid treatment.
^e^ Using only CPET values to maximal effort
^f^ Average z-score of aerobic capacity, hand-grip strength and lower body endurance.

Abbreviations: BMI, Body-Mass-Index; CI, Confidence Interval; PRR, Prevalence Rate Ratio.

**Supplemental Table 5, Association between physical fitness adjusted for lean-body-mass (LBM) and prevalence of adverse events from Poisson regression models, *N=154-157^a^***

|  | **Basic Model 0^b^** | | | **Adjusted Model 1^c^** | | | **Adjusted Model 2^d^** | | |
| --- | --- | --- | --- | --- | --- | --- | --- | --- | --- |
|  | PRR | 95%CI | p-value | PRR | 95%CI | p-value | PRR | 95%CI | p-value |
| **Events of any grade** | | | | | | | | | |
| Aerobic capacity  [watt/ kg LBM] | 0.73 | [0.67, 0.80] | <0.001 | 0.74 | [0.68, 0.82] | <0.001 | 0.74 | [0.67, 0.81] | <0.001 |
| Hand-grip strength [kg/ kg LBM] | 0.77 | [0.54, 1.11] | 0.160 | 1.00 | [0.67, 1.49] | 0.995 | 0.78 | [0.51, 1.19] | 0.248 |
| Lower body endurance  [5 repetitions/ kg LBM] | 0.25 | [0.09, 0.72] | 0.010 | 0.43 | [0.14, 1.32] | 0.141 | 0.41 | [0.13, 1.29] | 0.127 |
| **Events grades 2+** | | | | | | | | | |
| Aerobic capacity | 0.71 | [0.62, 0.80] | <0.001 | 0.72 | [0.63, 0.82] | <0.001 | 0.73 | [0.64, 0.83] | <0.001 |
| Hand-grip strength | 0.94 | [0.58, 1.54] | 0.811 | 1.35 | [0.79, 2.32] | 0.272 | 1.00 | [0.56, 1.76] | 0.994 |
| Lower body endurance | 0.17 | [0.04, 0.70] | 0.015 | 0.30 | [0.06, 1.41] | 0.129 | 0.31 | [0.06, 1.53] | 0.153 |
| **Events grades 3+** | | | | | | | | | |
| Aerobic capacity | 0.60 | [0.48, 0.74] | <0.001 | 0.59 | [0.47, 0.73] | <0.001 | 0.58 | [0.46, 0.73] | <0.001 |
| Hand-grip strength | 0.82 | [0.35, 1.92] | 0.236 | 1.08 | [0.42, 2.77] | 0.872 | 0.79 | [0.29, 2.13] | 0.647 |
| Lower body endurance | 0.23 | [0.02, 2.62] | 0.236 | 0.36 | [0.03, 5.04] | 0.446 | 0.44 | [0.03, 6.76] | 0.553 |

Footnotes:
^a^ n=154 for aerobic capacity, n=157 for hand-grip strength, n=154 for lower body endurance
^b^ Basic Model 0: adjusted for age, sex, time since diagnosis.
^c^ Adjusted Model 1: Model 0 + BMI.
^d^ Adjusted Model 2: Model 1 + cancer types, anthracycline dose, steroid dose, location of radiotherapy. We calculated the cumulative anthracyclines dose as doxorubicin isotoxic equivalent dose (mg/m^2^) and the cumulative steroid dose as prednisone equivalent dose (mg/m^2^). Patients who did not receive steroid or anthracycline treatment, received 0 mg/m^2^ as their dose; 6 participants who received steroids but had missing information on cumulative dose were imputed with the median dose of all participants with steroid treatment.

Abbreviations: BMI, Body-Mass-Index; CI, Confidence Interval; PRR, Prevalence Rate Ratio; LBM, Lean Body Mass.
