# Supplementary material for: Physical fitness and clinically assessed disease burden in long‐term childhood cancer survivors—The SURfit study
Source: Cancer. 2025 Aug 19;131(17):e70051. doi: 10.1002/cncr.70051 (PMC12365372; doi:10.1002/cncr.70051)
Supplement: Supplementary file 2 — Supplementary Material [file CNCR-131-e70051-s001.docx]

**Online Appendix: Detailed description of assessment and coding of disease burden within the SURfit study**

**Clinical assessments**

Aim 1:

All participants of the SURfit study underwent extensive medical examinations at the University Children’s Hospital Basel by trained staff, standardized in specific standard operating procedures. All assessments are described in detail in the published study protocol (1). Assessments were performed by trained staff (study nurses, physicians, or sports scientists) and based on standard operating procedures (SOPs). The assessments included patient history (demographic, cancer related, and lifestyle characteristics), medical record abstraction, clinical examination, blood analyses, physical fitness tests, electrocardiogram (ECG), oral glucose tolerance test (oGTT), pulmonary function test (Spirometry, only completed by patients having received chest radiation or bone marrow transplantation hence judged to be at “high-risk” for pulmonary function problems by the medical examination committee) and dual energy x-ray absorptiometry. We calculated the cumulative anthracyclines dose as doxorubicin isotoxic equivalent dose (mg/m^2^) (2) and the cumulative steroid dose as prednisone equivalent dose (mg/m^2^) (3), analog to previous SURfit publications (4). For the regression models, patients who did not receive steroid or anthracycline treatment, received 0 mg/m^2^ as their dose; 6 participants who received steroids but had missing information on cumulative dose were imputed with the median dose of all participants with steroid treatment.
Standardized and validated self-reported questionnaires were used for mental health (Brief Symptom Inventory (BSI-53) and fatigue (Checklist of Individual Strength (CIS)) assessment (5, 6). According to the CTCAE the grade 1 event *Depression* resp. *Anxiety* is defined as “mild depressive/anxiety symptoms”. Hence self-reported depressive/anxiety symptoms in the BSI (T-score ≥63 in the self-reported depression/anxiety subscale of the BSI-53 (7)) were classified as a grade 1 event. Patients with officially diagnosed depression/anxiety were classified as grade 1 if not treated and grade 2 if receiving treatment (medical or psychotherapy).

DXA:
Bone health and body composition were measured by Dual-Energy X-ray Absorptiometry (DXA) (Discovery A densitometer; Hologic, Bedford, MA). Quality assurance was checked and if needed calibrated before each measuring day according to manufacturers’ guidelines. Outcomes by DXA included total body, femoral neck, total hip and lumbar spine areal BMD by age- and sex-matched z-scores, as well as percent fat mass, absolute fat mass and lean body mass (total body and regional). Body composition estimation from DXA scan shows good precision with a 2–3% coefficient of variation (8).

Aim 2:
As part of the SURfit 1-year randomized-controlled exercise intervention trial physical activity was assessed at baseline (prior to start of the intervention), 3 months, 6 months, and 12 months. This paper uses only baseline data. As participants went on to be potentially included in an exercise intervention trial the inclusion criteria were specified accordingly: eligible participants who reported >4h of intense physical activities per week at baseline were excluded, because adding the target physical activity of the intervention on top of that might not be beneficial and difficult to implement. Physical fitness was assessed by trained staff (physicians or sports scientists) using maximal cardiopulmonary exercise testing (CPET), a hand-grip test (JAMAR Hydraulic Hand Dynamometer) and the 1-minute sit-to-stand test (STS).

CPET: *Aerobic capacity*
Peak performance (watt) was assessed by a continuous incremental cycling test (ergometer) to volitional exhaustion following the step protocol by Godfrey and colleagues in accordance with the international guidelines for exercise testing (9, 10). Heartrate was continuously measured. Ratings of perceived exertion and dyspnea were evaluated at peak exercise by means of a 0–10 Borg scale. The protocol started with 3 min rest, followed by a 3 min warm up (unloaded pedaling) and after that an incremental loading of +20 Watt/min until exhaustion. Peak performance (watt) was defined as the power maintained over the final 1-minute stage of the test, adding 5 watt for every 15 seconds of the non-finished stage. A recovery phase of 3 min (with pedaling at 0 resistance and 60 revolutions per minute (rpm)) followed. Participants were instructed to pedal at a target pedaling rate of 60-90 (rpm). If the participants were no longer able to continue cycling at a pedaling rate of ≥60 rpm the test was terminated. In our study exclusively Watt_max_ values were used as a proxy for cardio-respiratory fitness, as respiratory outcomes (VO2_max_) values were not obtained reliably and hence never published. A maximal incremental test is typically identified by a VO_2_ plateau despite increased work rate. This plateau is defined as a VO_2_ increase of less than 2 ml/kg/min for a 5–10% rise in intensity or below two standard deviations of the prior stage’s VO_2_ increase (11). However, not all individuals exhibit this plateau, thus, a test was considered maximal if at least three of the following criteria were met:

- Reaching predicted peak VO_2_ and/or maximal work rate (based on test, gender, and age).
- Attaining or exceeding 85% of predicted peak heart rate.
- Peak ventilation nearing estimated maximal voluntary ventilation (MVV = FEV_1_ × 35).
- A respiratory exchange ratio (RER) above 1.03 (adolescence) or 1.05 (adults).
- A Borg scale rating of 9–10.
- Examiner’s subjective assessment of a maximal effort.

For our main analysis, we included all CPET results (maximal and submaximal) for the association between *aerobic capacity* and adverse health outcomes. To investigate possible bias, we performed a sensitivity analysis including only maximal tests (n=128) which showed similar results (Supplemental Table 4).

Hand-grip Strength test: *Upper body strength*
Upper body strength was assessed by a *hand-grip strength* test with a JAMAR Hydraulic Hand Dynamometer (Lafayette Instrument®, Lafayette, USA) in a sitting position with flexed elbows and forearm on the table. We calculated the mean weight of three trials in kilograms reached by the dominant hand (12) per kilogram of bodyweight (kg/kg bodyweight).

1-min STS: *Lower body endurance*
The 1-min STS test measures a combination of strength, aerobic- and anaerobic capacity, and balance. Each participant performed 2 trials of the maximum number of sit-to-stand repetitions in 1 minute, 20 minutes apart, to account for a learning effect. Thus, the second trial was used in the analysis (1). The number of repetitions was divided by 5 to have a meaningful unit in the model. This rescaling allowed for effect estimates to be interpreted per 5 additional sit-to-stand repetitions per minute, which represents a meaningful clinical difference compares to a single repetition (the minimal important difference is 5 repetitions) (13).

**Classification according to the Common Terminology of Adverse Events**

Every adverse health event or symptom registered after the date of the first childhood cancer diagnosis until baseline in the above described clinical assessments was classified according to the preferred terms (PT) within each system organ class (SOC) according to the Common Terminology of Adverse Events (CTCAE) manual version 5 (14). This newest version was published in November 2017 hence may account for discrepancies in chronic condition counts compared to earlier studies such as the St. Jude lifetime Cohort Study (2013) (15). The official amendments from version 4 to 5 can be found online: <https://ctep.cancer.gov/protocoldevelopment/electronic_applications/docs/CTCAE_v5.0.xlsx> We retained the original categories and names for all SOC, with the following exceptions: *Cardiac*- and *vascular* *disorders* were combined into one SOC *cardiovascular disorders*; *General disorders and administration site conditions* was renamed *General symptoms: pain, fatigue, edema; Investigations* was renamed *laboratory, imaging and functional findings*. These adjustments were made to ensure a better understanding of the according disorders. Each adverse event was further graded from 1 to 4 according to the definitions in the CTCAE manual as grade 1 (mild, requiring observation only), grade 2 (moderate with minimal or noninvasive intervention indicated and limited instrumental Activities of Daily Life (ADL) possible), grade 3 (severe event with limited ability of Self-Care ADL), or grade 4 (life-threatening event requiring urgent intervention). Grade 5, death, was not applicable in this study including survivors only. If an outcome occurred multiple times (such as two independent fractures) each outcome was calculated as a separate event. If an outcome changed in severity (such as increasing depressive symptoms) the outcome was recorded once and graded by its most severe presentation.

Relapse(s) and metastases were considered the primary disease and not classified as a CTCAE event; only second primary cancer diagnoses were recorded as an adverse event. Consequences of the primary tumor resection such as amputation or enucleation were classified as a CTCAE event, as they directly impact ADL. Genetic disorders, although present since birth, were included as a CTCAE event if diagnosed after the primary cancer diagnosis as they imply further adverse outcomes. Reported allergic rhinitis was not recorded as a CTCAE due to the genetic and environmental disease origin.

Pre-defined criteria for specific adverse events:

For the following variables no applicable criteria were defined in the CTCAE v5, hence, the following predefined criteria and grading were specified:

1) Mental health (cut-offs analogue to the previous SURfit publication by Deng et al. (16)):

*Anxiety*: categorized as grade 1 if T-score ≥63 in the self-reported anxiety subscale of the BSI-53 (7). Patients with officially diagnosed anxiety were classified grade 1 if not treated and grade 2 if receiving treatment (medical or psychotherapy).

*Depression*: categorized as grade 1 if T-score ≥63 in the self-reported depression subscale of the BSI-53 (7). Patients with officially diagnosed depression were classified grade 1 if not treated and grade 2 if receiving treatment (medical or psychotherapy).

*Fatigue*: categorized as grade 1 if self-reported CIS severity of fatigue raw score was ≥27, and grade 2 if ≥35 (17).

*Insomnia*: classified as grade 1 if the patient reported “slight” difficulty falling asleep, grade 2 if the difficulty was “mostly” or “strong”, and grade 3 if it was “very strong”.

2) Musculoskeletal health:

*Generalized muscle weakness*: categorized as grade 1 if patients scored <60% of the predicted value of age and sex standardized references in the CPET (8), hand-grip test (12), or STS (18).

*Peripheral sensory neuropathy*: categorized as grade 1 if vibration perception with tuning fork was ≤6/8 in the upper extremities (metacarpal), and ≤5/8 in the lower extremities (metatarsal) (19). Reflexes/missing reflexes were not counted as an adverse event.

*Osteoporosis*: age and sex matched z-scored were used (analogue to the previous SURfit publication by Zürcher et al. (20)). If a z-score below the reported cut-offs defined in the CTCAE guidelines was reported for any of the measured body sites (total body, lumbar spine, hip or femoral neck) the adverse event was classifyed with the respective grade.

*Fractures*: adhered to CTCAE definition whereby ankle, hip, spinal, or wrist fractures are classified separately and must be used prior to applying the generalized event “fracture”. Hence “fractures” refers to a fracture of all other sites. Grading of fractures is based on symptom severity, not the mechanism of injury, so we are unable to differentiate between fractures sustained from low-impact versus high-impact activities.

3) Metabolic and endocrine health:

*Endocrine disorders*: Hypogonadism was categorized only if an official diagnosis was reported in the medical record. Patients were designated grade 1 if untreated and grade 2 if undergoing treatment. Clinical abnormalities, such as Tanner stages, were not documented as CTCAE events. Delayed puberty, as per CTCAE, was assessed based on breast development; grade 2 if hormone replacement therapy (HRT) was not warranted and grade 3 if HRT was received. Additionally, female patients with menarche after 15 years of age were classified as having "delayed puberty," with analog grading determined according to having received HRT.

*Metabolism and nutrition disorders*: Patients with an underweight BMI (≤ 18) were classified grade 1. Reportings of taking supplements such as Vitamin D, Magnesium or Calcium were not classified as adverse event, as a medical indication was not guaranteed.

4) Other:

*Hearing impaired*: Whenever exact threshold values of hearing loss were not reported, impaired hearing was classified as grade 1.

*Thromboembolic events*: Patients with cranial thromboembolic events were classified as “Nervous system disorders, stroke” and graded based on the severity of neurological and potentially life-threatening consequences. Patients with peripheral thromboembolic events were classified as “Vascular disorders, thromboembolic event”.

**REFERENCES**

1. Rueegg CS, Kriemler S, Zuercher SJ, Schindera C, Renner A, Hebestreit H, et al. A partially supervised physical activity program for adult and adolescent survivors of childhood cancer (SURfit): study design of a randomized controlled trial [NCT02730767]. BMC Cancer. 2017;17(1):822.

2. Children's Oncology Group Long-Term Follow-Up Guidelines for Survivors of Childhood, Adolescent, and Young Adult Cancers2023; 6:[1-180 pp.]. Available from: <http://www.survivorshipguidelines.org/>.

3. Inaba H, Pui CH. Glucocorticoid use in acute lymphoblastic leukaemia. Lancet Oncol. 2010;11(11):1096-106.

4. Schindera C, Zürcher SJ, Jung R, Boehringer S, Balder JW, Rueegg CS, et al. Physical Fitness and Modifiable Cardiovascular Disease Risk Factors in Survivors of Childhood Cancer: A Report From the SURfit Study. Cancer. 2021;127:1690-8.

5. Derogatis LR, Melisaratos N. The Brief Symptom Inventory: an introductory report. Psychol Med. 1983;13(3):595-605.

6. Vercoulen JH, Swanink CM, Fennis JF, Galama JM, van der Meer JW, Bleijenberg G. Dimensional assessment of chronic fatigue syndrome. J Psychosom Res. 1994;38(5):383-92.

7. Franke GH. BSI. Brief Symptom Inventory - Deutsche Version. 2000.

8. Tothill P, Avenell A, Love J, Reid DM. Comparisons between Hologic, Lunar and Norland dual-energy X-ray absorptiometers and other techniques used for whole-body soft tissue measurements. Eur J Clin Nutr. 1994;48(11):781-94.

9. Godfrey S. Exercise tests in assessing children with lung or heart disease. Thorax. 1970;25(2):258.

10. Society AT, Physicians ACoC. ATS/ACCP Statement on cardiopulmonary exercise testing. Am J Respir Crit Care Med. 2003;167(2):211-77.

11. Sheehan JM, Rowland TW, Burke EJ. A comparison of four treadmill protocols for determination of maximum oxygen uptake in 10- to 12-year-old boys. Int J Sports Med. 1987;8(1):31-4.

12. Werle S, Goldhahn J, Drerup S, Simmen BR, Sprott H, Herren DB. Age- and gender-specific normative data of grip and pinch strength in a healthy adult Swiss population. Journal of Hand Surgery, European Volume. 2009;34(1):76-84.

13. Radtke T, Puhan MA, Hebestreit H, Kriemler S. The 1-min sit-to-stand test--A simple functional capacity test in cystic fibrosis? J Cyst Fibros. 2016;15(2):223-6.

14. National Cancer Institute: U.S. Department of Health and Human Services; 2021 [Available from: <https://ctep.cancer.gov/>.

15. Hudson MM, Ness KK, Gurney JG, Mulrooney DA, Chemaitilly W, Krull KR, et al. Clinical ascertainment of health outcomes among adults treated for childhood cancer. JAMA. 2013;309(22):2371-81.

16. Deng WH, Zürcher SJ, Schindera C, Jung R, Hebestreit H, Bänteli I, et al. Effect of a 1-year physical activity intervention on quality of life, fatigue, and distress in adult childhood cancer survivors-A randomized controlled trial (SURfit). Cancer. 2024.

17. Blaauwbroek R, Bouma MJ, Tuinier W, Groenier KH, de Greef MH, Meyboom-de Jong B, et al. The effect of exercise counselling with feedback from a pedometer on fatigue in adult survivors of childhood cancer: a pilot study. Support Care Cancer. 2009;17(8):1041-8.

18. Strassmann A, Steurer-Stey C, Lana KD, Zoller M, Turk AJ, Suter P, et al. Population-based reference values for the 1-min sit-to-stand test. International Journal of Public Health. 2013;58(6):949-53.

19. Hilz MJ, Axelrod FB, Hermann K, Haertl U, Duetsch M, Neundörfer B. Normative values of vibratory perception in 530 children, juveniles and adults aged 3-79 years. Journal of Neurological Sciences. 1998;159(2):219-25.

20. Zürcher SJ, Jung R, Monnerat S, Schindera C, Eser P, Meier C, et al. High impact physical activity and bone health of lower extremities in childhood cancer survivors: A cross-sectional study of SURfit. International Journal of Cancer. 2020;147(7):1845-54.
